# MicroRNA Sequencing of Serum Exosomes Reveals miR205-5p as an Anti-Fibrogenic Factor Against Intestinal Fibrosis in Crohn’s Disease

**DOI:** 10.3390/ijms26083778

**Published:** 2025-04-17

**Authors:** Ying Xie, Mieke van Daelen, Rebecca Park, Isabella Almaraz, Lindsey Fontenot, Florian Rieder, Wendy Ho, Berkeley Limketkai, David Q. Shih, Minjie Wei, Yiling Li, Hon Wai Koon

**Affiliations:** 1 Vatche and Tamar Manoukian Division of Digestive Diseases, David Geffen School of Medicine, University of California Los Angeles, Los Angeles, CA 90095, USA; yingxie2019@hotmail.com (Y.X.); miekevandaelen@g.ucla.edu (M.v.D.); rebeccakp@g.ucla.edu (R.P.); ialmaraz@g.ucla.edu (I.A.); lindsey.fontenot01@gmail.com (L.F.); wwho@mednet.ucla.edu (W.H.); blimketkai@mednet.ucla.edu (B.L.); 2 Department of Gastroenterology and Endoscopy, The First Hospital of China Medical University, Shenyang 110001, China; 3 Department of Gastroenterology, Hepatology, and Nutrition, Digestive Diseases Institute, Department of Inflammation and Immunity, Lerner Research Institute, Program for Global Translational Inflammatory Bowel Diseases, Cleveland Clinic Foundation, 9500 Euclid Avenue, Cleveland, OH 44195, USA; riederf@ccf.org; 4 F. Widjaja Foundation, Inflammatory Bowel & Immunobiology Research Institute, Cedars-Sinai Medical Center, Los Angeles, CA 90048, USA; david10021@gmail.com; 5 Department of Pharmacology, School of Pharmacy, China Medical University, Shenyang 110001, China; weiminjiecmu@163.com

**Keywords:** fibrosis, miRNA, exosome

## Abstract

More than half of Crohn’s disease patients eventually develop intestinal strictures. Intestinal fibrosis is the excessive deposition of the extracellular matrix that obstructs intestinal movement. There is no approved medication to treat intestinal stricture. The roles of serum exosomal miRNAs in intestinal fibrosis are unknown. Serum exosomal miRNA sequencing was performed on samples of healthy donors and stricturing CD (CDS) patients. CDS patient-derived primary intestinal fibroblasts (CD-HIFs), CDS patient-derived serum exosomes (CDSE), human peripheral blood mononuclear cells (PBMCs), human colonic tissues, and mouse models of intestinal fibrosis were used. CDS patients had significantly lower serum exosomal miR205-5p levels than non-CDS patients and healthy donors. CDS patients had reduced miR205-5p expression in PBMCs. miR205-5p reduced its target Zinc finger E-box-binding homeobox 1 (ZEB1) and collagen protein expression in CDSE-treated CD-HIFs. In mouse models of intestinal fibrosis, overexpression of miR205-5p inhibited intestinal fibrosis, which was overcome by Zeb1 overexpression. Elafin, a human anti-fibrogenic protein, induced miR205-5p in intestinal fibroblasts. Inhibition of miR205-5p reversed the anti-fibrogenic effects of elafin in mice. Low serum exosomal anti-fibrogenic miR205-5p levels were associated with intestinal strictures in CD patients. miR205-5p can mediate the anti-fibrogenic effect of elafin by inhibiting ZEB1 and collagen expression.

## 1. Introduction

Crohn’s disease (CD) affects approximately 0.3% of Americans [1]. About 30–50% of CD patients may eventually develop an intestinal stricture, with intestinal fibrosis being a significant component [2,3]. Intestinal fibrosis is characterized by excessive deposition of extracellular matrix (ECM), particularly collagen, which leads to clinically apparent bowel obstruction. These intestinal strictures often necessitate hospitalization. While procedures such as endoscopic balloon dilation and strictureplasty can alleviate mild, short-segment bowel narrowing, more severe and complex bowel obstructions may require surgical resection [4]. Surgical resection is considered a last resort due to its adverse impact on the patient’s quality of life. Currently, no pharmacological treatments are available for intestinal fibrosis, illuminating the urgent need for developing anti-fibrogenic therapies that reduce dependence on surgical interventions.

Host responses are critically involved in intestinal fibrosis development. Exosomes are cell-derived extracellular vesicles of 30–150 nm in size that mediate cell–cell communication [5]. The nucleic acid, protein, and lipids in exosomes may regulate inflammatory bowel diseases (IBD), including CD [6]. Some of the serum exosomal microRNAs (miRNAs) correlate with disease activity in IBD patients [7,8].

Our recent report indicated that serum contains pro-fibrogenic factors that induce collagen expression in intestinal fibroblasts [9]. Only serum exosomes from stricturing CD (CDS) patients, but not healthy donors and non-stricturing CD (CDNS) patients, induced collagen expression in intestinal fibroblasts [9]. Notably, serum exosomes from CDS patients (CDSE) only induced collagen expression in intestinal fibroblasts but not epithelial cells [10]. CDSE had lower miR205-5p levels than serum exosomes from healthy donors and CDNS patients [9]. miR205-5p was shown to suppress cardiac and pulmonary fibrosis [11,12]. These findings suggest that miR205-5p has an anti-fibrogenic property. However, the cellular source of miR205-5p and its effect on intestinal fibrosis were not determined.

Elafin, a human antimicrobial peptide and protease inhibitor, is a promising potential therapeutic agent against intestinal fibrosis as it inhibits Zinc finger E-box-binding homeobox 1 (ZEB1) and collagen expression in intestinal fibroblasts as well as ameliorates intestinal fibrosis in multiple mouse models of intestinal fibrosis [10]. miR205-5p is known to target ZEB1 [13,14]. ZEB1 is a transcriptional factor for collagen expression [15]. These findings prompted further investigation into the role of miR205-5P in intestinal fibrosis and its association with the anti-fibrogenic effects of elafin.

We hypothesize that serum exosomal miR205-5p levels can reflect the presence of intestinal strictures in CD patients. Additionally, miR205-5p is a potential therapeutic target for intestinal fibrosis. This study provided further insight into the anti-fibrogenic mechanism of elafin against intestinal fibrosis.

## 2. Results

### 2.1. CDS Patients Had Lower Serum Exosomal miR205-5p Levels than Non-CDS Patients

**Figure 1:**
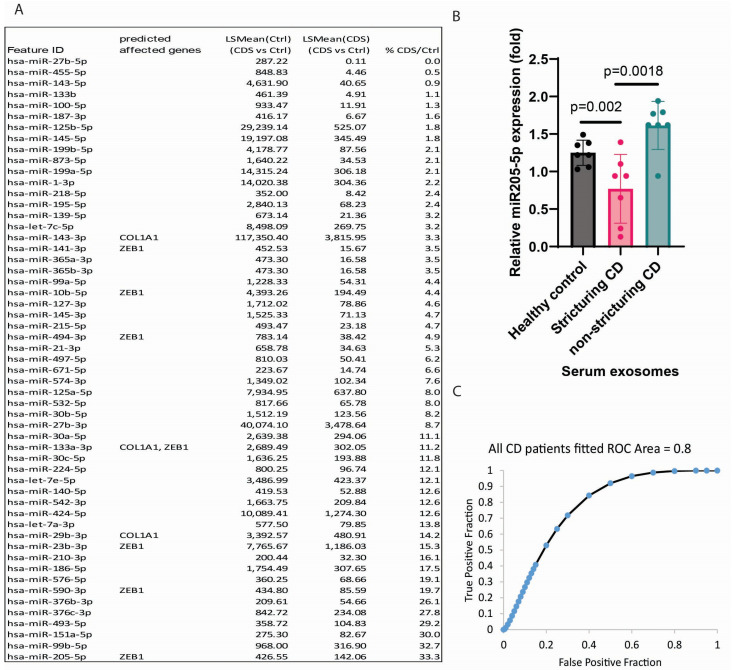
CDS patients have low serum exosomal levels of miR205-5p. (**A**) A list of serum exosomal miRNAs. Their levels in CDS patients were 33.3% or lower compared to those in healthy donors. LSMeans = least-squares means. Some miRNAs are predicted to target ZEB1 and COL1A1. Samples were pooled from 12 CDS patients and 12 healthy donors. (**B**) Real-time RT-PCR of serum exosomes. Relative miR205-5p expression was normalized to RNU1A1 expression. In total, 7 healthy donors, 7 CDS patients, and 9 CDNS patients were included. Mean ± SD. (**C**) The ROC curve and the AUC value show the accuracy of using serum exosomal miR205-5p for intestinal stricture indication among 16 CD patients. The analysis was performed using an online ROC curve calculator: http://www.rad.jhmi.edu/jeng/javarad/roc/JROCFITi.html, (accessed on 8 April 2025). AUC = 0.8 indicates moderate accuracy of serum exosomal miR205-5p in indicating intestinal strictures in CD patients.

Our group had previously identified a correlation between serum exosomes and intestinal strictures in CD patients [9]. To explore the miRNA cargo in serum exosomes in CDS patients, we performed miRNA sequencing and detected 176 miRNAs in the serum exosomes (Table 1). In CDS patients, there were 55 serum exosomal miRNAs with lower levels than those in healthy donors (Figure 1A). Seven down-regulated serum exosomal miRNAs in CDS patients were predicted to affect ZEB1, while several others were predicted to affect the collagen type I alpha 1 (COL1A1) gene (Figure 1A).

miR205 emerged as a key miRNA due to our prior findings linking the miR205-3p inhibitor’s promotion of collagen expression in intestinal fibroblasts [9]. Our miRNA sequencing indicated that serum exosomal miR205-5p levels in CDS patients were just 33.3% relative to those in healthy donors (Figure 1A). Real-time RT-PCR validation experiments confirmed our previous PCR array finding [9], as serum exosomal miR205-5p levels in CDS patients were significantly lower than those in healthy donors and CDNS patients (Figure 1B).

### 2.2. Low Serum Exosomal miR205-5p Levels May Indicate Intestinal Strictures in CD Patients

To evaluate whether serum exosomal miR205-5p levels indicate intestinal strictures, we determined their accuracy with receiver operating characteristic (ROC) curve analysis. Serum exosomal miR205-5p levels exhibited a moderate correlation with the presence of intestinal strictures in CD patients (area under curve/AUC = 0.8) (Figure 1C). Therefore, the low serum exosomal miR205-5p level is moderately accurate in indicating intestinal strictures in CD patients.

### 2.3. CDS Patient-Derived Peripheral Blood Mononuclear Cells (PBMCs) Had Low miR205-5p Expression

**Figure 2:**
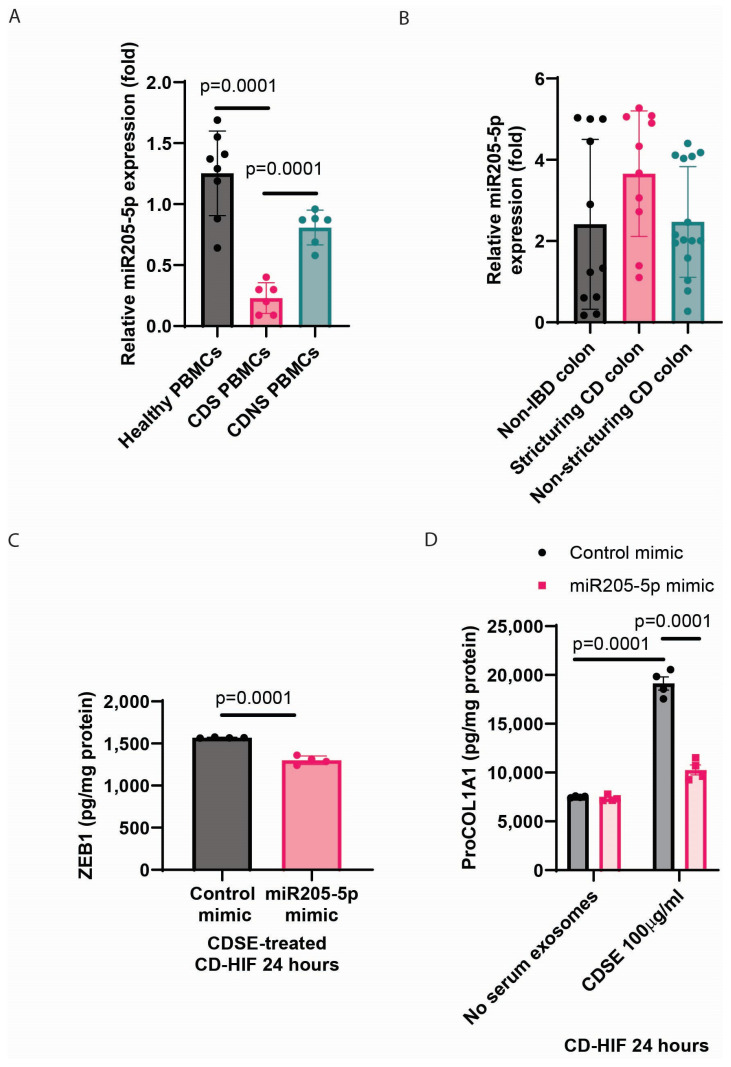
CDS-PBMCs had low expression of anti-fibrogenic miR205-5p. (**A**) Real-time RT-PCR of PBMCs. Relative intracellular miR205-5p expression was normalized to RNU1A1 expression. A total of 8 healthy donors, 6 CDS patients, and 6 CDNS patients were included. Mean ± SD. One-way ANOVA. (**B**) Real-time RT-PCR of colonic tissues. Relative miR205-5p expression was normalized to 18S rRNA expression. A total of 11 healthy donors, 10 CDS patients, and 15 CDNS patients were included. Mean ± SD. One-way ANOVA. Note: the transcript number of these colonic tissue samples was low (Ct values > 35). (**C**,**D**) CD-HIFs were transfected with (30 pmol/mL) miR205-5p mimic via (3 μL/mL) Lipofectamine 3000 (L3000001, ThermoFisher, Waltham, MA, USA), followed by treatment with and without 100 μg/mL CDSE for 24 h. ZEB1 and ProCOL1A1 protein expression in cell lysates were determined by ELISA. Mean ± SD. (**C**) *T*-test. (**D**) One-way ANOVA.

PBMCs express serum exosomal miRNAs [16]. To identify the cellular source of miR205-5p, real-time RT-PCR of PBMCs from various patients was performed. CDS patients had significantly lower intracellular miR205-5p expression in PBMCs than healthy donors and CDNS patients (Figure 2A). Still, there was no difference in the colonic miR205-5p expression among non-IBD, CDS, and CDNS patients (Figure 2B). This trend suggests that the lower serum exosomal miR205-5p may be correlated with the lower miR205-5p expression in the PBMCs of CDS patients.

### 2.4. miR205-5p Inhibited Collagen Expression Only in a Fibrogenic Environment

As miR205-5p was shown to affect ZEB1 expression [13], transfection of an miR205-5p mimic significantly reduced ZEB1 protein expression in CD patient-derived human intestinal fibroblasts (CD-HIFs) treated with CD patient-derived serum exosomes (CDSE) (Figure 2C). Consistent with our previous reports [9,10], CDSE, a pro-fibrogenic factor, stimulated collagen Pro-collagen I alpha 1 ProCOL1A1 expression in CD-HIFs and supported the CDS environment (Figure 2D). Transfection of miR205-5p mimic abolished CDSE-induced ProCOL1A1 protein expression in CD-HIFs but did not affect ProCOL1A1 protein expression in basal conditions (Figure 2D).

### 2.5. Lentiviral miR205-5p Overexpression Ameliorated Intestinal Fibrosis in Mice

Most intestinal strictures in CD patients occur in the ileum, but some also affect the colon [17]. SAMP1/YitFc mice have ileal fibrosis [18]. Trinitrobenzene sulfonic acid (TNBS)-treated mice have colonic fibrosis [19]. These two mouse models of intestinal fibrosis cover the disease locations of CD strictures.

**Figure 3:**
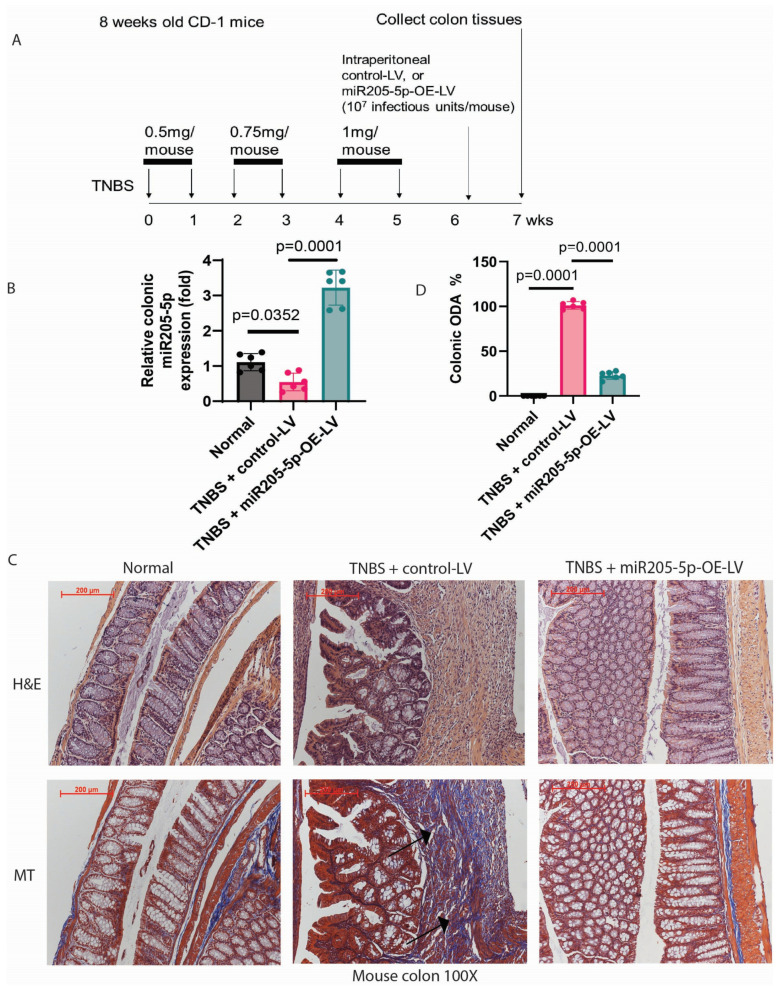
Lentiviral miR205-5p overexpression ameliorated colonic fibrosis in TNBS-treated mice. (**A**) Experimental plan of TNBS colonic fibrosis induction and miR205-5p-overexpressing lentivirus treatment in mice. (**B**) Real-time RT-PCR of colonic tissues on the last day of the experiment. mmu-miR205-5p expression was normalized to RNU1A1 expression. Mean ± SD. *T*-test. The arrow points to the fibrotic region with excessive collagen deposition. (**C**) H&E and Masson Trichrome staining of colonic tissues. The blue color indicates ECM deposition. miR205-5p lentiviral overexpression reduced colonic ECM deposition. Six mice per group. (**D**) Colonic ODA. Mean ± SD. One-way ANOVA.

Repeated intracolonic injections of TNBS cause colonic fibrosis in mice [10]. TNBS-treated mice were intraperitoneally injected with miR205-5p-overexpressing lentivirus to study the anti-fibrogenic effect of miR205-5p in colonic fibrosis (Figure 3A). As expected, TNBS treatment diminished colonic miR205-5p expression (Figure 3B), causing substantial colonic injury (as shown by hematoxylin and eosin staining) and ECM deposition (as indicated by Masson Trichrome staining) (Figure 3C) and increased colonic overall disease activity (ODA) (Table 2). ODA is an objective indicator of multiple fibrosis and inflammation markers in the intestines [10]. Lentiviral miR205-5p overexpression increased colonic miR205-5p expression (Figure 3B), reduced colonic injury and fibrosis (Figure 3C), and lowered ODA (Table 2).

**Figure 4:**
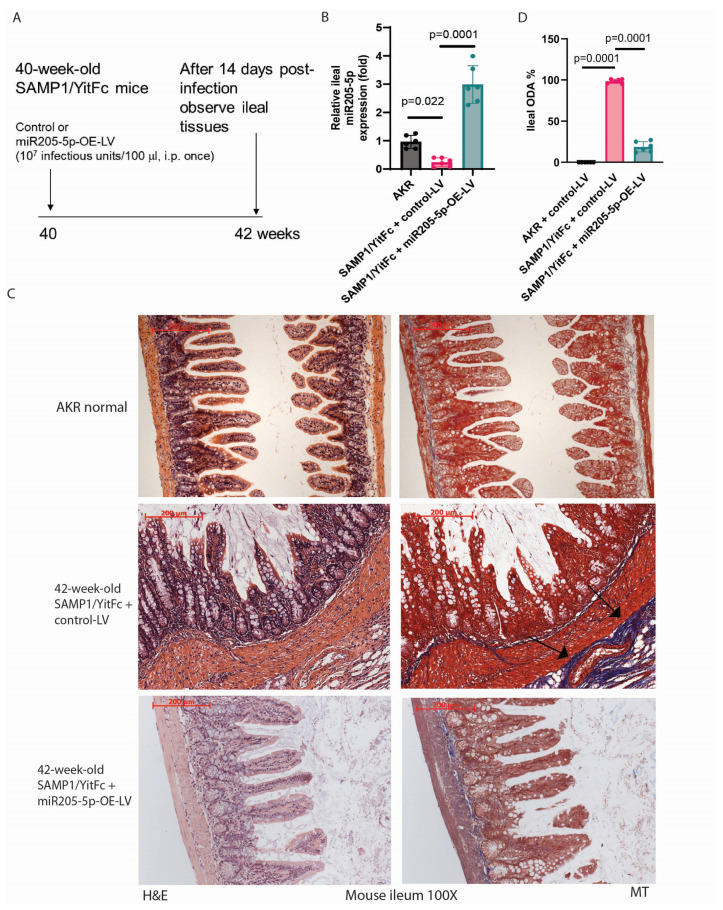
Lentiviral miR205-5p overexpression ameliorated ileal fibrosis in SAMP1/YitFc mice. (**A**) Experimental plan of miR205-5p-overexpressing lentivirus treatment in SAMP1/YitFc mice with ileal fibrosis. (**B**) Real-time RT-PCR of ileal tissues on the last day of the experiment. mmu-miR205-5p expression was normalized to RNU1A1 expression. Mean ± SD. *T*-test. (**C**) H&E and Masson Trichrome staining of ileal tissues. The blue color indicates ECM deposition. miR205-5p lentiviral overexpression reduced ileal ECM deposition. Six mice per group. The arrow points to the fibrotic region with excessive collagen deposition. (**D**) Ileal ODA. Mean ± SD. One-way ANOVA.

On the other hand, SAMP1/YitFc mice develop CD-like spontaneous ileitis and ileal fibrosis around 40 weeks of age [18,20]. Like the TNBS model, SAMP1/YitFc mice were intraperitoneally injected with miR205-5p-overexpressing lentivirus to study the anti-fibrogenic effect of miR205-5p in ileal fibrosis (Figure 4A). Compared to their normal parental control strain AKR mice, SAMP1/YitFc mice had increased ileal ODA with reduced ileal miR205-5p expression (Table 3 and Figure 4B). Lentiviral miR205-5p overexpression increased ileal miR205-5p expression (Figure 4B). As expected in [10], SAMP1/YitFc mice had more ileal injury and ECM deposition than AKR mice (Figure 4C). Consistent with the TNBS model, lentiviral miR205-5p overexpression reduced ileal injury and fibrosis (Figure 4C) and lowered ODA in SAMP1/YitFc mice (Figure 4D and Table 3).

Table 2 and Table 3 show detailed information about colonic and ileal histology scores, fibrosis scores, and mRNA expression of several relevant inflammatory and fibrogenic genes. These parameters were used to calculate the ODA [10]. Lentiviral miR205-5p overexpression consistently reduced mRNA expression of colonic and ileal collagen (collagen type I alpha 2/Col1a2 and collagen type III alpha 1/Col3a1), Zeb1, and fibroblast markers (alpha-smooth muscle actin/Acta2 and vimentin/Vim) in TNBS-treated and SAMP1/YitFc mice, respectively.

### 2.6. Zeb1 Overexpression Abolished the Anti-Fibrogenic Effect of miR205-5p

**Figure 5:**
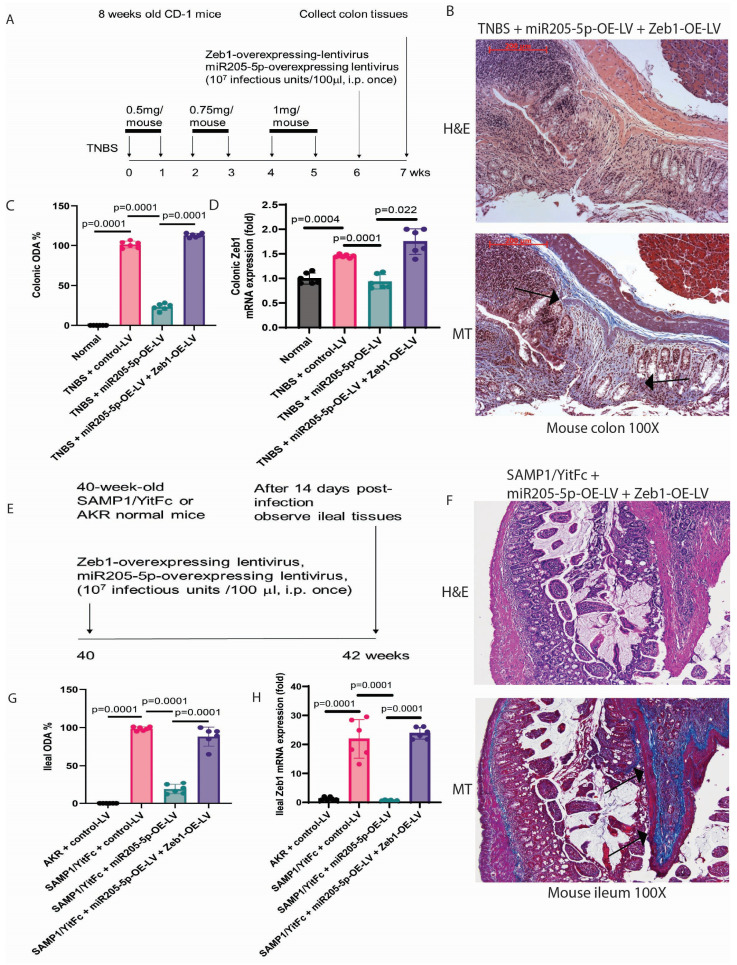
Anti-fibrogenic effect of miR205-5p was abolished by Zeb1 overexpression in fibrotic mice. (**A**) Experimental plan of miR205-5p-overexpressing and Zeb1-overexpressing lentivirus treatment in TNBS-treated mice with colonic fibrosis. (**B**) H&E and Masson Trichrome staining of colonic tissues. The blue color indicates ECM deposition. Lentiviral co-overexpression of miR205-5p and Zeb1 maintained excessive colonic ECM deposition. Six mice per group. (**C**) Colonic ODA of TNBS-treated mice. Mean ± SD. One-way ANOVA. (**D**) Colonic Zeb1 mRNA expression. Mean ± SD. One-way ANOVA. (**E**) Experimental plan of miR205-5p-overexpressing and Zeb1-overexpressing lentivirus treatment in SAMP1/YitFc mice with ileal fibrosis. (**F**) H&E and Masson Trichrome staining of ileal tissues. The blue color indicates ECM deposition. Lentiviral co-overexpression of miR205-5p and Zeb1 maintained excessive ileal ECM deposition. Six mice per group. Scale bar = 100 microns. (**G**) Ileal ODA of SAMP1/YitFc mice. Mean ± SD. One-way ANOVA. (**H**) Ileal Zeb1 mRNA expression. Mean ± SD. One-way ANOVA. The arrow points to the fibrotic region with excessive collagen deposition.

The pro-fibrogenic property of Zeb1 was revealed in our previous report as Zeb1 short hairpin RNA (shRNA)-lentivirus reduced colonic fibrosis in TNBS-treated and ileal fibrosis in SAMP1/YitFc mice [10]. The anti-fibrogenic effect of lentiviral miR205-5p overexpression against colonic fibrosis in TNBS-treated mice was abolished by lentiviral Zeb1 overexpression (Figure 5A,B and Table 2). Similarly, the anti-fibrogenic effect of lentiviral miR205-5p overexpression against ileal fibrosis in SAMP1/YitFc mice was abolished by lentiviral Zeb1 overexpression (Figure 5E,F and Table 3), suggesting the miR205-5p-Zeb1 anti-fibrogenic pathway. For both mouse models of intestinal fibrosis, the ODA-lowering and Zeb1-inhibitory effects of lentiviral miR205-5p overexpression were reversed by lentiviral Zeb1 overexpression (Figure 5C,D,G,H).

### 2.7. Elafin Inhibited ZEB1 and Collagen Expression via miR205-5p Induction in Intestinal Fibroblasts

**Figure 6:**
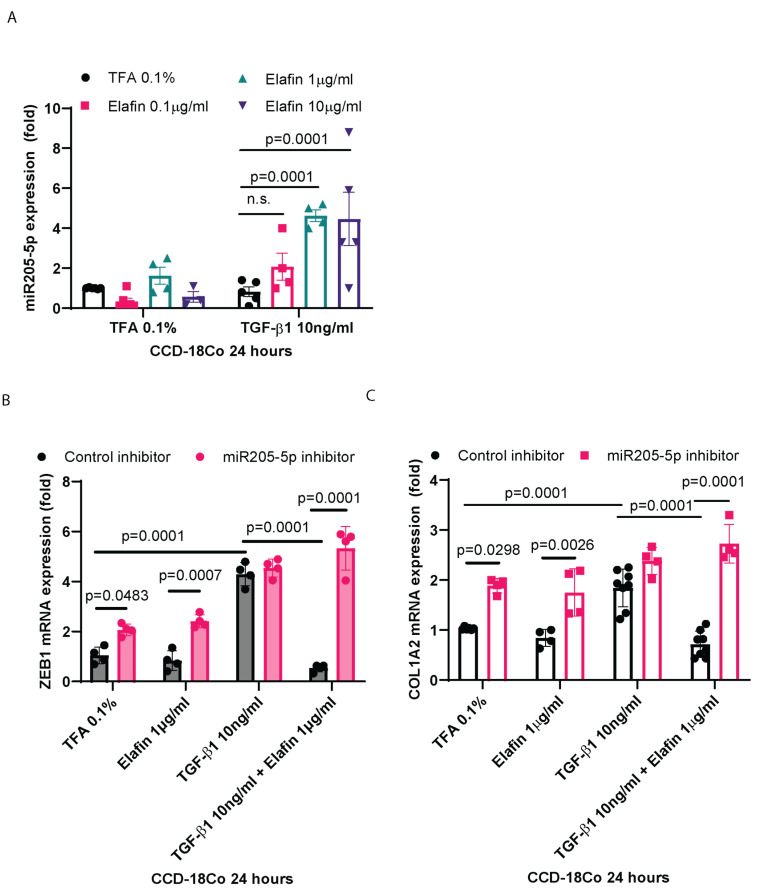
Elafin-mediated inhibition of ZEB1 and COL1A2 mRNA expression was reversed by miR205-5p inhibition in intestinal fibroblasts. (**A**) Real-time RT-PCR. Colonic CCD-18Co fibroblasts were treated with elafin and TGF-β1 for 24 h. miR205-5p expression was normalized to RNU1A1 expression. Elafin at 1–10 μg/mL induced miR205-5p expression in the TGF-β1-treated CCD-18Co fibroblasts. Mean ± SD. One-way ANOVA. Four experiments. (**B**,**C**) Real-time RT-PCR. Colonic CCD-18Co fibroblasts were treated with elafin, TGF-β1, and 30 pmol/mL control inhibitor or miR205-5p inhibitor for 24 h. Relative ZEB1 and COL1A2 mRNA expression was normalized to 18S rRNA. Mean ± SD. One-way ANOVA. Four experiments.

Elafin is an anti-fibrogenic protein that inhibits collagen expression via ZEB1 suppression in intestinal fibroblasts [10]. Interestingly, elafin increased miR205-5p expression in transforming growth factor beta 1 (TGF-β1)-treated human colonic CCD-18Co fibroblasts but had no effect under basal conditions (Figure 6A). Unlike the CDSE treatment, the pro-fibrogenic TGF-β1 treatment avoided the bias of low miR205-5p in the CDSE [9]. CCD-18Co fibroblasts provided an alternative human intestinal fibroblast model for comparison [9].

TGF-β1 induced collagen ZEB1 and COL1A2 mRNA expression in CCD-18Co fibroblasts (Figure 6B,C). Elafin reduced ZEB1 and COL1A2 mRNA expression in TGF-β1-treated CCD-18Co fibroblasts, which was reversed by the addition of miR205-5p inhibitor (Figure 6B,C). These findings suggested that the anti-fibrogenic effect of elafin was mediated by the induction of ZEB1-targeting miR205-5p in intestinal fibroblasts.

### 2.8. Elafin Mediated Anti-Fibrogenic Effects via miR205-5p in Mice with Intestinal Fibrosis

**Figure 7:**
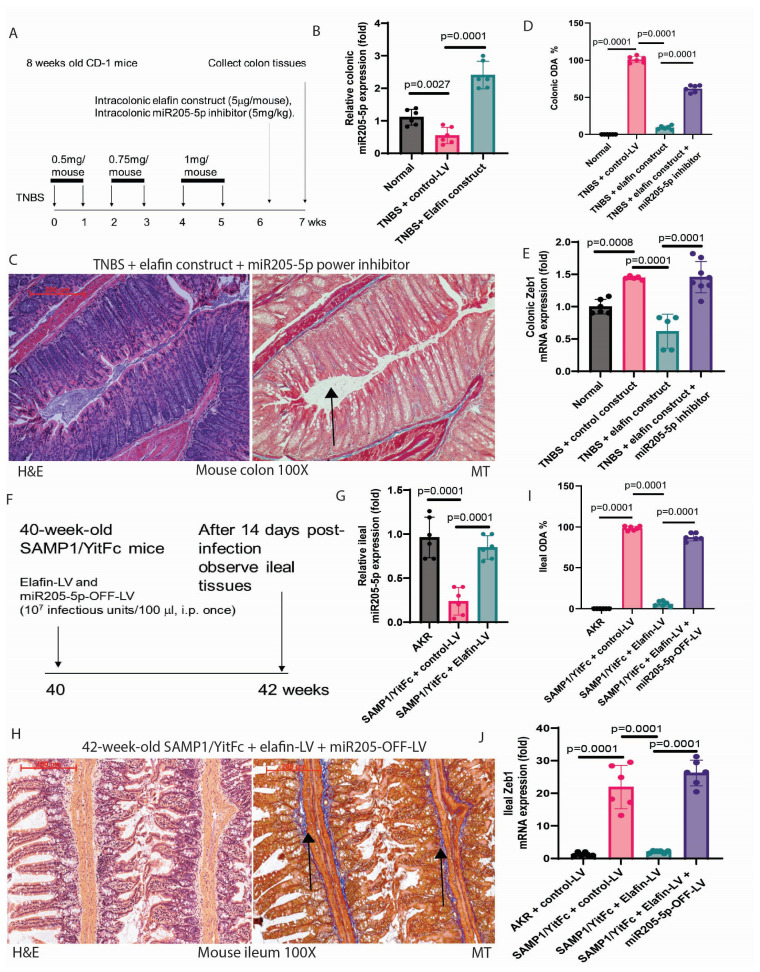
The anti-fibrogenic effect of elafin overexpression was reversed by miR205-5p inhibition. (**A**) Experimental plan of intracolonic elafin-overexpressing construct and miR205-5p inhibitor treatment in TNBS-treated mice with colonic fibrosis. (**B**) Real-time RT-PCR. Colonic miR205-5p expression, which was normalized to RNU1A1 expression. Mean ± SD. One-way ANOVA. (**C**) H&E and Masson Trichrome staining of colonic tissues. The blue color indicates ECM deposition. Intracolonic elafin overexpression and miR205-5p inhibition maintained excessive colonic ECM deposition. Six mice per group. (**D**) Colonic ODA. Mean ± SD. One-way ANOVA. (**E**) Colonic Zeb1 mRNA expression. Mean ± SD. One-way ANOVA. (**F**) Experimental plan of elafin-overexpressing and miR205-5p inhibitory lentivirus treatment in SAMP1/YitFc mice with ileal fibrosis. (**G**) Real-time RT-PCR. Ileal miR205-5p expression, which was normalized to RNU1A1 expression. Mean ± SD. One-way ANOVA. (**H**) H&E and Masson Trichrome staining of ileal tissues. The blue color indicates ECM deposition. Systemic elafin overexpression and miR205-5p inhibition maintained excessive ileal ECM deposition. Six mice per group. (**I**) Ileal ODA. Mean ± SD. One-way ANOVA. (**J**) Ileal Zeb1 mRNA expression. Mean ± SD. One-way ANOVA. The arrow points to the fibrotic region with excessive collagen deposition.

The inhibitory effects of intracolonic elafin overexpression against colonic fibrosis in TNBS-treated mice and lentiviral elafin overexpression against ileal fibrosis in SAMP1/YitFc mice were reported previously [10]. Intracolonic transfection with an elafin-overexpressing construct inhibited colonic fibrosis and reduced the colonic ODA from 100% to 9% in TNBS-treated mice [10]. The same elafin overexpression treatment increased colonic miR205-5p expression (Figure 7A,B). On the other hand, intracolonic injection of miR205-5p inhibitor abolished the anti-fibrogenic effect of elafin in the TNBS-treated mice (Figure 7C). It returned the colonic ODA to 59% (Figure 7D and Table 2).

Intraperitoneal injection of an elafin-overexpressing lentivirus inhibited ileal fibrosis and reduced ileal ODA from 100% to 6% in SAMP1/YitFc mice [10]. The same elafin overexpression treatment restored ileal miR205-5p expression (Figure 7F,G). On the other hand, the intraperitoneal injection of miR205-5p inhibitory lentivirus abolished the anti-fibrogenic effect of elafin (Figure 7H) and increased the ileal ODA to 85% (Figure 7I and Table 3).

Elafin effectively decreased colonic and ileal Zeb1 mRNA expression in TNBS-treated mice and SAMP1/YitFc mice [10]. Inhibition of miR205-5p increased colonic and ileal Zeb1 mRNA expression to levels close to those of positive control groups (Figure 7E,J and Table 2 and Table 3). Overall, elafin increased intestinal miR205-5p expression, leading to amelioration of intestinal fibrosis with reduced intestinal Zeb1 and collagen mRNA expression in mice.

### 2.9. miR205-5p Manipulation Did Not Consistently Affect the Body Weight of Mice with Intestinal Fibrosis

Similar to our previous study [10], TNBS treatment did not significantly affect body weight in the last two weeks of the experiments (Table 4), while the average body weight of fibrotic SAMP1/YitFc mice was also comparable to that of non-fibrotic AKR mice (Table 5). Lentiviral miR205-5p overexpression caused a reduction in body weight by 15% and 8% in TNBS-treated and SAMP1/YitFc mice, respectively (Table 4 and Table 5). Compared to their positive control group, the differences in miR205-5p-mediated weight loss were statistically insignificant.

Elafin overexpression slightly increased body weight in TNBS-treated mice and SAMP1/YitFc mice, but the differences were statistically insignificant (Table 4 and Table 5). The body weight of TNBS-treated mice transfected with intracolonic elafin overexpressing construct was not affected by intracolonic miR205-5p inhibitor treatment (Table 4). Similarly, SAMP1/YitFc mice infected with elafin-overexpressing lentivirus were not affected by lentiviral miR205-5p inhibition (Table 5). Overall, body weight in mice was unaffected by the presence of intestinal fibrosis, elafin overexpression, and manipulation of miR205-5p.

## 3. Discussion

Our previous and current studies validated low serum exosomal miR205-5p levels in CDS patients [9], which may be caused by the low intracellular miR205-5p expression in their PBMCs (Figure 2A). Both studies also confirmed that the lack of miR205 was adequate to drive fibrogenesis because inhibitors of miR205-5p and miR205-3p increased collagen expression in intestinal fibroblasts (Figure 6C) [9]. This finding underscored the miRNA-dependent interactions between immune cells and fibroblasts in the development of intestinal fibrosis.

The novelty of this study is the demonstration of the anti-fibrogenic roles of miR205-5p in intestinal fibrosis with the support of mouse study data. Unsurprisingly, serum exosomal miRNAs are involved in diseases. Several years ago, our group discovered a negative correlation between serum exosomal miR181b-5p and miR210-3p and fasting blood glucose among men with type II diabetes [16]. Elafin stimulated miR181b-5p and miR210-3p expression in adipocytes, leading to increased leptin production in adipocytes [16]. Increased leptin production can promote satiety, reduce food consumption, and restore insulin sensitivity [21]. Consequently, elafin mediated anti-obesity and anti-diabetic effects via serum exosomal miR181b-5p and miR210-3p in high-fat diet-treated male mice [16]. Both our previous and current studies indicated that serum exosomal miRNAs are mediators of diseases and therapeutic targets of elafin in various diseases [9,16].

Direct application of miRNAs to treat diseases is challenging. Clinical trials have been conducted, but off-target effects remain a safety concern [22]. None of the miRNA-based therapies have been approved by the US Food and Drug Administration (FDA). It is unlikely for miR205-5p mimic to become a direct therapeutic agent in the foreseeable future. However, it may still be a clinically relevant biomarker for the presence of intestinal strictures or the efficacies of anti-fibrogenic agents against intestinal fibrosis.

Serum exosomal miRNAs have been implicated in fibrosis in other organs [23,24]. Some exosomal miRNAs are associated with IBD [25]. However, the applications of serum exosomal miRNAs as biomarkers in clinical settings are still challenging unless there are standardized exosome isolation methods, robust miRNA detection, and large-scale clinical trials.

The immunoregulatory effects of miR205-5p were inconsistent among models as lentiviral miR205-5p overexpression significantly reduced ileal tumor necrosis factor (Tnf) and EGF-like module containing mucin-like hormone receptor (Emr1) mRNA expression in SAMP/YitFc mice (Table 3) without affecting colonic Tnf and Emr1 mRNA expression in TNBS-treated mice (Table 2). As shown by the miRNA target prediction database (miRDB), TNF (a proinflammatory cytokine) and EMR1 (a macrophage marker) are not direct targets of miR205-5p (Supplementary Materials). Interestingly, miR205 may promote healing [26,27]. 

The limitation of this study is the scale of validation. The miRNA sequencing detected many CDS-relevant miRNAs (Figure 1A). Some of these serum exosomal miRNAs (such as miR29) may affect intestinal fibrosis [28]. However, validating their targets and functions in intestinal fibrosis is tedious and expensive.

As shown by the miRDB, miRNAs can affect many targets (Supplementary Materials). For example, cadherin-11 (CDH11) is a putative target of miR205-5p (miRDB data in Supplementary Materials). CDH11 was shown to mediate fibrogenic activities in intestinal fibrosis [29]. However, the mechanistic relationship between miR205-5p and CDH11 has not been experimentally validated.

As indicated by our RNA sequencing, ZEB1 is the only up-regulated miR205-5p target in CD colonic strictures [9]. Colonic ZEB1 mRNA expression was not affected in UC and CDNS patients [10]. ZEB1 was mechanistically proven to mediate collagen expression in both intestinal fibroblasts and mouse models [10]. To further explore the anti-fibrogenic mechanism of elafin, only miR205-5p is worth extended evaluation using cell culture and animal models considering ZEB1 and collagen regulation and relevance to CD stricture [9,10].

As stated in Qiagen’s miRNA handbook, miRNA quantitative PCR is unreliable for measuring the performance of miRNA inhibitors because miRNA inhibitors form stable complexes with miRNA targets but do not degrade them. The miR205-5p expression with lentiviral miR205-OFF-LV could not be accurately determined with miR205-5p PCR assays. Therefore, it is unfeasible to show the miR205-5p expression in mice with miR205-5p inhibition.

In conclusion, low serum exosomal miR205-5p levels may be linked to low miR205-5p expression in immune cells in CDS patients. miR205-5p is anti-fibrogenic as it inhibits ZEB1 and collagen expression in intestinal fibroblasts. Anti-fibrogenic elafin can induce miR205-5p expression in intestinal fibroblasts, which can target ZEB1 and collagen expression and subsequently inhibit intestinal fibrosis. The findings of this study provided new insights into the mechanism of intestinal fibrosis.

## 4. Materials and Methods

### 4.1. Human Serum Samples and Colonic Tissues

Serum samples of healthy donors and CD patients were prospectively collected from the University of California Los Angeles (UCLA) from 2012 to 2015. The physicians requested the medically indicated blood collection. The pooled sera from 12 stricturing CD patients were used for preparing serum exosomes (CDSE) [9,10]. Serum exosomes were prepared by total exosome isolation reagent (#4478360, ThermoFisher, Waltham, MA USA) and quantified by bicinchoninic acid (BCA) protein assay (#23225, ThermoFisher), as described previously [10].

Frozen colonic tissue samples of CD patients were collected from the Cedars-Sinai Medical Center (CSMC) prospectively during the surgical resection of diseased tissues from 2010 to 2014 and cryopreserved until this study. Frozen human colonic tissues were used for comparing gene expression in stricturing CD and non-stricturing CD patients [9,10].

Inclusion criteria: IBD, intestinal strictures, and colon cancer were diagnosed by gastroenterologists as described previously [9].

Exclusion criteria: Pregnant women, concurrent acute infections (cytomegalovirus infection, *C. difficile* infection, and *tuberculosis*), and malignant conditions were excluded because these factors may affect CD disease activity [30,31,32]. Minors under age 18 were excluded due to the complexity of obtaining consent.

Baseline characteristics of patients are shown in Table 6.

Serum exosomal miRNA sequencing and bioinformatic analysis were performed by the UCLA Technology Center for Genomics and Bioinformatics (TCGB).

### 4.2. Intestinal Fibroblast Culture

Crohn’s disease patient-derived human intestinal fibroblasts (CD-HIFs) were prepared from intestinal tissues in stricturing CD patients [9,10]. Serum-starved CD-HIFs were pretreated with 100 μg/mL CDSE to support the CDS environment and induce collagen expression [9,10]. Baseline characteristics of donors are shown in Table 6.

Human colonic CCD-18Co fibroblasts (CRL-1459, ATCC) were cultured in MEM media with 10% fetal bovine serum and 1% penicillin-streptomycin. All cells were grown to 80% confluence and then switched to serum-free media overnight for experiments. Serum-starved CCD-18Co were pretreated with either 0.1% trifluoroacetic acid (TFA) as a vehicle or 10 ng/mL transforming growth factor beta 1 (TGF-β1), followed by incubation with elafin (#AS-61641, Anaspec, Fremont, CA, USA) [10]. Pro-fibrogenic TGF-β1 is known to increase collagen expression in CCD-18Co fibroblasts [9].

At the end of the experiments, cells were lysed with radioimmunoprecipitation assay (RIPA) buffer (#89900, ThermoFisher) containing protease and phosphatase inhibitor cocktail (PPC1010, Sigma, Burlington, MA, USA) for ELISA. We utilized ELISA to measure protein levels of ProCOL1A1 (DY6220-05, R&D Systems, Minneapolis, MN, USA) and ZEB1 (MBS774017, MyBioSource, San Diego, CA 92195-3308 USA) in cell lysates [10]. Alternatively, cells were lysed with Qiagen’s, Germantown, MD, USA, RLT buffer for RNA experiments [10].

### 4.3. Peripheral Blood Mononuclear Cells (PBMCs)

PBMCs from healthy donors, stricturing CD patients, and non-stricturing CD patients were cultured in RPMI1640 media containing 10% exosome-depleted fetal bovine serum (A2720803, ThermoFisher) and 1% penicillin-streptomycin [10]. Baseline characteristics are shown in Table 6.

### 4.4. Animal Experiments

Mice were blindly randomized and assigned to cages by animal facility staff. They were then housed in the UCLA animal facility under standard environmental conditions. All interventions were performed during the light cycle.

(*TNBS*) Eight-week-old male and female outbred CD-1 mice (#022, Charles River, Wilmington, MA, USA) were injected with trinitrobenzene sulfonic acid (TNBS) solution in 30% ethanol via enema weekly six times (weeks 0, 1, 2, 3, 4, and 5) [10]. The 30% ethanol was used to help the TNBS penetrate the colonic mucosa. Colonic fibrosis typically develops one week after the last injection (week 6). The non-fibrotic control group was treated with six weekly injections of 30% ethanol via enema.

Some TNBS-treated mice received a single intracolonic injection of 5 μg/mouse of either control construct (PS100001) or elafin-overexpressing construct (RC203136) from Origene via InvivoJetPEI transfection reagent (201-10G, Polyplus) on the ninth day after the last TNBS injection. Some TNBS-treated mice received a single intracolonic injection of 5 nmol of mmu-miR-205-5p power inhibitor (GeneGlobe ID: YI04101506, Cat. No.: 339131, Qiagen, Germantown, MD, USA). Some TNBS-treated mice had intraperitoneal injections of control lentivirus (PS100064V), miR205-5p-overexpressing lentivirus (LV7-mm40214, Abmgood, Richmond, BC, Canada), and Zeb1-overexpressing lentivirus (MR223095L2V) from Origene, Inc. on the ninth day after the last TNBS injection. Colonic tissues were collected two weeks after the 6th TNBS injection.

(*SAMP1/YitFc*) Forty-week-old male and female SAMP1/YitFc mice (#009355, Jackson Laboratories, Bar Harbor, ME, USA) were used [10]. This model develops chronic ileitis with pre-existing ileal fibrosis around 40 weeks of age. Forty-week-old AKR mice (#000648, Jackson Laboratories) were used as a parental non-fibrotic control strain [20].

Control lentivirus (PS100064V, Origene, Rockville, MD, USA), miR205-5p-overexpressing lentivirus (LV7-mm40214, Abmgood), miR205-5p inhibiting lentivirus (LV7-mm30328, Abmgood), elafin-overexpressing lentivirus (RC203136L1V, Origene), and Zeb1-overexpressing lentivirus (MR223095L2V, Origene) were injected into SAMP1/YitFc mice intraperitoneally once at 40 weeks of age. Ileal tissues were collected for analyses at 42 weeks of age.

### 4.5. Histological Evaluations

Intestinal tissue injury was evaluated with Hematoxylin and Eosin (H&E) staining, while ECM deposition was identified by Masson Trichrome (MT) staining. H&E- and MT-stained microphotographs were recorded at multiple locations and scored blindly by two investigators. The chronic intestinal injury was scored in terms of mucosal transformation (0/3/6), round cell infiltration in the lamina propria mucosa (0–3), goblet cell death (0/1), tela submucosa fibrosa (0/1), and granuloma (0/1). These parameters result in a total score (0–12) [33]. In addition, intestinal fibrosis was scored on a scale of normal, mild, moderate, and severe (0–3) [34].

### 4.6. Quantitative Real-Time RT-PCR

Total RNA was isolated by an RNeasy kit (#74104, Qiagen).

For the detection of mRNAs, RNA was reverse transcribed into cDNA (#4368813, ThermoFisher). PCR reactions were run with cDNA, iTaq Universal SYBR Green Supermix (1725120, Bio-Rad, Hercules, CA, USA), and TaqMan real-time PCR assays in a Bio-Rad CFX384 system [10]. Catalog numbers of PCR assays of tested human and mouse genes were reported previously [10]. Relative mRNA expression of genes was normalized to either human 18S rRNA or mouse Gapdh expression.

miRNA was isolated by an miRNeasy kit (#217084, Qiagen).

For the detection of miR205-5p, RNA was reverse transcribed into cDNA using an miRCURY Locked Nucleic Acid (LNA) reverse transcription (RT) kit (#339340, Qiagen). PCR reactions were run with cDNA, miRCURY LNA SYBR Green PCR kit (#339345, Qiagen), and hsa-miR205-5p miRCURY LNA miRNA PCR assay (GeneGlobe Id: YP00204487, Catalog Number: 339306, Qiagen). This assay detects both human and mouse miR205-5p. Relative miR205-5p expression was normalized to RNU1A1 expression. RNU1A1 miRCURY LNA miRNA PCR assay (GeneGlobe Id: YP00203909, Catalog Number: 339306, Qiagen).

After normalization with endogenous control genes, relative mRNA quantification was performed by comparing test and control groups. The fold changes are expressed as 2^∆∆Ct^. Fold-change values greater than one indicate a positive or an up-regulation. Conversely, fold-change values less than one indicate a negative or down-regulation.

### 4.7. Calculation of Overall Disease Activities (ODA)

Histology scores, fibrosis scores, and mRNA expression of several fibrosis-related and inflammation-related genes commonly reported by our and other intestinal fibrosis research groups were converted into percentages to help compare between groups and models of intestinal fibrosis. ODA is the average value of these parameters [10].

### 4.8. Statistical Analysis

Results were expressed as mean +/− standard deviation (SD). We utilized GraphPad Prism 10 to perform multiple-group comparisons using ordinary one-way Analyses of Variance (ANOVAs) with Tukey’s post hoc tests and two-group comparisons using Student’s *t*-tests. The *p* values of statistical significance are shown in each figure or table.

## Figures and Tables

**Table 1:** A total of 176 serum exosomal miRNAs were detected in both CDS patients and healthy donors.

hsa-miR-27b-5p	hsa-let-7g-5p	hsa-miR-25-3p
hsa-miR-455-5p	hsa-miR-369-5p	hsa-miR-148b-3p
hsa-miR-143-5p	hsa-miR-376a-3p	hsa-miR-103a-3p
hsa-miR-133b	hsa-miR-151a-3p	hsa-miR-185-5p
hsa-miR-100-5p	hsa-miR-20b-5p	hsa-miR-425-3p
hsa-miR-187-3p	hsa-miR-338-3p	hsa-miR-335-5p
hsa-miR-125b-5p	hsa-miR-132-3p	hsa-miR-320a-3p
hsa-miR-145-5p	hsa-miR-19b-3p	hsa-miR-1277-5p
hsa-miR-199b-5p	hsa-miR-7-5p	hsa-miR-107
hsa-miR-873-5p	hsa-miR-21-5p	hsa-miR-484
hsa-miR-199a-5p	hsa-miR-199a-3p	hsa-miR-128-3p
hsa-miR-1-3p	hsa-miR-199b-3p	hsa-miR-376b-5p
hsa-miR-218-5p	hsa-let-7f-5p	hsa-miR-376c-5p
hsa-miR-195-5p	hsa-miR-98-5p	hsa-miR-122-5p
hsa-miR-139-5p	hsa-let-7b-5p	hsa-miR-223-3p
hsa-let-7c-5p	hsa-miR-191-5p	hsa-miR-146a-5p
hsa-miR-143-3p	hsa-miR-222-3p	hsa-miR-151b
hsa-miR-141-3p	hsa-miR-24-3p	hsa-miR-486-5p
hsa-miR-365a-3p	hsa-miR-374a-5p	hsa-miR-503-5p
hsa-miR-365b-3p	hsa-miR-30e-5p	hsa-miR-423-5p
hsa-miR-99a-5p	hsa-miR-152-3p	hsa-miR-542-5p
hsa-miR-10b-5p	hsa-miR-30d-5p	hsa-miR-432-5p
hsa-miR-127-3p	hsa-miR-26a-5p	hsa-miR-92a-3p
hsa-miR-145-3p	hsa-let-7d-5p	hsa-miR-183-5p
hsa-miR-215-5p	hsa-miR-144-3p	hsa-miR-361-3p
hsa-miR-494-3p	hsa-miR-101-3p	hsa-miR-589-5p
hsa-miR-21-3p	hsa-miR-142-5p	hsa-miR-17-5p
hsa-miR-497-5p	hsa-miR-126-3p	hsa-miR-182-5p
hsa-miR-671-5p	hsa-miR-451a	hsa-miR-664a-5p
hsa-miR-574-3p	hsa-miR-20a-5p	hsa-miR-337-5p
hsa-miR-125a-5p	hsa-miR-19a-3p	hsa-miR-130b-3p
hsa-miR-532-5p	hsa-miR-431-5p	hsa-miR-4732-5p
hsa-miR-30b-5p	hsa-miR-144-5p	hsa-miR-744-5p
hsa-miR-27b-3p	hsa-miR-361-5p	hsa-miR-382-5p
hsa-miR-30a-5p	hsa-miR-10a-5p	hsa-miR-1307-3p
hsa-miR-133a-3p	hsa-miR-194-5p	hsa-miR-4454
hsa-miR-30c-5p	hsa-miR-450b-5p	hsa-miR-223-5p
hsa-miR-224-5p	hsa-miR-409-3p	hsa-miR-363-3p
hsa-let-7e-5p	hsa-miR-3615	hsa-miR-320b
hsa-miR-140-5p	hsa-miR-339-5p	hsa-miR-320c
hsa-miR-542-3p	hsa-miR-23a-3p	hsa-miR-625-3p
hsa-miR-424-5p	hsa-miR-148a-3p	hsa-miR-320d
hsa-let-7a-3p	hsa-let-7i-5p	hsa-miR-584-5p
hsa-miR-29b-3p	hsa-miR-340-5p	hsa-miR-339-3p
hsa-miR-23b-3p	hsa-miR-93-5p	hsa-miR-181a-5p
hsa-miR-210-3p	hsa-miR-22-3p	hsa-let-7d-3p
hsa-miR-186-5p	hsa-miR-15b-5p	hsa-miR-328-3p
hsa-miR-576-5p	hsa-miR-369-3p	hsa-miR-629-5p
hsa-miR-590-3p	hsa-miR-326	hsa-miR-660-5p
hsa-miR-376b-3p	hsa-miR-130a-3p	hsa-miR-32-5p
hsa-miR-376c-3p	hsa-miR-378a-3p	hsa-miR-485-3p
hsa-miR-493-5p	hsa-miR-654-3p	hsa-miR-22-5p
hsa-miR-151a-5p	hsa-miR-29c-3p	hsa-miR-1301-3p
hsa-miR-99b-5p	hsa-miR-16-5p	hsa-miR-539-3p
hsa-miR-205-5p	hsa-miR-221-3p	hsa-miR-323a-3p
hsa-miR-29a-3p	hsa-miR-192-5p	hsa-miR-323b-3p
hsa-miR-28-3p	hsa-miR-142-3p	
hsa-let-7a-5p	hsa-miR-425-5p	
hsa-miR-342-3p	hsa-miR-140-3p	
hsa-miR-16-2-3p	hsa-miR-126-5p	

**Table 2:** Colonic histology scores, fibrosis scores, gene expression profiles, and overall disease activities of TNBS-treated mice. *p*-values of comparison between groups are shown below the ODA values. n = 6 mice per group.

		HS	FS	Col1a2	Col3a1	Zeb1	Vim	Acta2	Tnf	Emr1	ODA%
Colonic		Score	Score	mRNA	mRNA	mRNA	mRNA	mRNA	mRNA	mRNA	Mean ± sd
**Normal**	mean	0.00	0.00	0.89	0.90	0.98	1.17	1.10	1.18	1.05	**0**
	sd	0.00	0.00	0.17	0.40	0.28	0.28	0.15	0.29	0.61	
	%	0.0	0.0	0.0	0.0	0.0	0.0	0.0	0.0	0.0	
**TNBS + control LV**	mean	8.33	2.43	1.95	2.51	1.45	2.51	2.39	1.75	2.39	**100 ± 3.26**
	sd	1.30	0.49	0.41	0.32	0.25	1.08	1.02	0.31	0.95	
	%	100	100	100	100	100	100	100	100	100	
**TNBS + miR205-5p-OE-LV**	mean	5.14	1.14	1.11	1.05	0.93	1.09	0.85	1.28	1.68	**23 ± 4.48**
	sd	1.35	0.83	0.73	0.66	0.12	0.49	0.44	0.44	0.34	
	%	62	47	20	10	0	0	0	18	47	
compared to TNBS + control LV											**0.0001**
**TNBS + miR205-5p-OE-LV + Zeb1-OE-LV**	mean	10.60	2.67	2.79	3.33	1.61	2.10	2.20	1.73	1.97	**113 ± 3.01**
	sd	0.59	0.81	0.36	0.69	0.20	0.65	0.33	0.84	0.46	
	%	127	110	180	151	136	69	85	96	69	
compared to TNBS + control LV											**0.0001**
**TNBS + elafin construct + control inhibitor**	mean	5.60	0.60	0.41	0.48	0.58	1.13	0.79	1.24	0.56	**11 ± 2.42**
	sd	1.67	0.59	0.12	0.20	0.18	0.29	0.09	0.47	0.11	
	%	46	0	0	0	0	0	0	62	0	
compared to TNBS + control construct											**0.0001**
**TNBS + elafin construct + miR205-5p inhibitor**	mean	8.80	2.20	1.20	1.28	1.45	1.95	1.82	1.52	1.80	**59 ± 3.06**
	sd	2.50	0.49	0.20	0.24	0.13	0.22	0.14	0.34	0.08	
	%	102	88	24	24	66	62	56	49	56	
compared to TNBS +elafin-LV											**0.0001**

**Table 3:** Ileal histology scores, fibrosis scores, gene expression profiles, and overall disease activities of SAMP1/YitFc mice. *p*-Values of comparison between groups are shown below the ODA values. n = 6 mice per group.

Ileal		HS	FS	Col1a2	Col3a1	Zeb1	Vim	Acta2	Tnf	Emr1	ODA%
		Score	Score	mRNA	mRNA	mRNA	mRNA	mRNA	mRNA	mRNA	Mean ± sd
**42-week-old AKR + control-LV**	mean	0.00	0.00	1.12	1.11	1.10	1.10	0.80	1.07	1.10	**0**
	sd	0.00	0.00	0.46	0.49	0.49	0.49	0.92	0.51	0.49	
	%	0	0	0	0	0	0	0	0	0	
**42-week-old SAMP1Yit/Fc + control-LV**	mean	11.80	2.80	20.33	23.57	21.90	482.75	2.31	2.05	4.42	**100 ± 3.06**
	sd	0.49	0.49	6.36	11.38	5.76	93.42	1.16	0.79	2.37	
	%	100	100	100	100	100	100	100	100	100	
compared to AKR											
**42-week-old SAMP1Yit/Fc + miR205-5p-OE-LV**	mean	5.30	1.67	1.27	1.70	0.68	1.59	1.23	0.36	2.00	**18 ± 2.12**
	sd	1.47	0.49	0.16	0.45	0.09	0.07	3.37	0.14	1.02	
	%	45	60	1	3	0	0	28	0	27	
compared to SAMP control-LV											**0.0001**
**42-week-old SAMP1Yit/Fc + miR205-5p-OE-LV + Zeb1-OE-LV**	mean	9.70	2.30	19.40	20.65	25.60	389.49	2.62	1.81	4.16	**92 ± 5.49**
	sd	3.67	0.49	3.45	0.16	3.84	21.48	0.83	1.02	1.12	
	%	82	82	95	87	118	81	120	76	92	
compared to SAMP miR205-5p-OE-LV											**0.0001**
**42-week-old SAMP1Yit/Fc + elafin-LV**	mean	2.00	0.80	0.74	1.73	2.42	44.19	0.65	0.53	0.25	**7 ± 4.26**
	sd	0.49	0.73	0.33	0.42	0.65	18.92	0.25	0.11	0.07	
	%	109	102	0	124	131	-19	0	0	0	
compared to SAMP control-LV											**0.0001**
**42-week-old SAMP1Yit/Fc + elafin-LV + miR205-OFF-LV**	mean	11.00	2.00	15.17	17.32	28.73	302.36	2.79	2.66	3.18	**85 ± 1.44**
	sd	1.22	0.81	0.91	1.51	0.92	28.03	1.27	7.79	2.52	
	%	93	71	73	72	133	63	131	162	63	
compared to SAMP elafin-LV											**0.0001**

**Table 4:** Average body weight of TNBS-treated mice. n = 6 mice per group.

Change in Body Weight in the Last 2 Weeks	
Normal	118%
TNBS + control-LV	115%
TNBS + miR205-5p-LV	100%
TNBS + Zeb1-OE-LV	105%
TNBS + Elafin construct	122%
TNBS + Elafin construct + miR-205 inhibitor	124%

**Table 5:** Average body weight of SAMP1/YitFc mice. n = 6 mice per group.

Changes in Body Weight from Week 40 to Week 42	
AKR normal control	100%
SAMP1/YitFc + control-LV	99%
SAMP1/YitFc + miR205-OE-LV	91%
SAMP1/YitFc + Elafin-LV	106%
SAMP1/YitFc + Elafin-LV + miR205-OFF-LV	105%
SAMP1/YitFc + miR205-OE-LV + Zeb1-OE-LV	104%

**Table 6:** Baseline characteristics of frozen human colonic tissue samples, human serum samples, CD-HIFs, and PBMCs.

**Colonic Tissue Samples** **(Mean ± sd)**		**Non IBD**	**CD Without** **Stricture**	**CD with** **Stricture**
Age at collection		62 ± 13.9	45 ± 19	36 ± 23.6
Gender (% male)		73	62	50
Histology score		2.6 ± 1.9	8.8 ± 3.7	8.6 ± 4.6
Simple colitis activity score		N/A	N/A	N/A
Harvey Bradshaw Index		N/A	7.5 ± 1.2	5 ± 1.9
% of biologics		0	50	40
% of 6MP or steroid		0	66	33
Duration of disease (years)		26 ± 3	8±3	18 ± 3
n		40	28	15
**SERUM Samples (mean ± sd)**		**Non IBD**	**CD Without** **Stricture**	**CD with** **Stricture**
Serum elafin levels (pg/mL)		7939 ± 791	7042±520	11,263 ± 1818
Age at collection		46 ± 12	34 ± 11	40±13
Gender (% male)		42	40	60
Harvey Bradshaw Index		N/A	3.7	5
Partial Mayo Score		N/A	N/A	N/A
% of biologics		N/A	40	46
% of 6MP or steroid		N/A	33	42
Duration of disease (years)		N/A	11 ± 1.4	11 ± 2.3
n		12	33	20
**CD-HIF** Patient	1	2	3	4
Age	41	45	66	68
Gender	Male	Female	Male	Female
Disease	CD stricture	CD stricture	CD stricture	CD stricture
Disease location	ileum	ileum	colon	colon
**PBMCs**		**Normal**	**CDNS**	**CDS**
Mean age (years)		33	38	34
Male sex (%)		50%	50%	50%
Caucasian		50%	50%	50%
Other ethnicity		50%	50%	50%
Ileum involvement		0%	50%	50%
Ileocolonic involvement		0%	50%	50%
n		8	8	8

## Data Availability

All data, analytical methods, and study materials will be available to other researchers. Please contact Hon Wai Koon.

## Supplementary Materials

The following supporting information can be downloaded at https://www.mdpi.com/article/10.3390/ijms26083778/s1.
