# Supplementary material for: MicroRNA Sequencing of Serum Exosomes Reveals miR205-5p as an Anti-Fibrogenic Factor Against Intestinal Fibrosis in Crohn’s Disease
Source: Int J Mol Sci. 2025 Apr 17;26(8):3778. doi: 10.3390/ijms26083778 (PMC12028105; doi:10.3390/ijms26083778)
Supplement: Supplementary file 1 [file ijms-26-03778-s001.zip › ijms-3520869-supplementary.pdf]

There are 737 predicted targets for hsa-miR-205-5p in miRDB.

Target Detail	Target Rank	Target Score	miRNA Name	Gene Symbol	Gene Description
Details	1	100	hsa-miR-205-5p	MOSMO	modulator of smoothened
Details	2	99	hsa-miR-205-5p	BICC1	BicC family RNA binding protein 1
Details	3	99	hsa-miR-205-5p	PPP2R2D	protein phosphatase 2 regulatory subunit Bdelta
Details	4	99	hsa-miR-205-5p	CHN1	chimerin 1
Details	5	99	hsa-miR-205-5p	CDK19	cyclin dependent kinase 19
Details	6	99	hsa-miR-205-5p	RAB11FIP1	RAB11 family interacting protein 1
Details	7	98	hsa-miR-205-5p	BTBD3	BTB domain containing 3
Details	8	98	hsa-miR-205-5p	LPCAT1	lysophosphatidylcholine acyltransferase 1
Details	9	98	hsa-miR-205-5p	RBM47	RNA binding motif protein 47
Details	10	98	hsa-miR-205-5p	AAK1	AP2 associated kinase 1
Details	11	97	hsa-miR-205-5p	PTPRJ	protein tyrosine phosphatase, receptor type J
Details	12	97	hsa-miR-205-5p	MAP3K13	mitogen-activated protein kinase kinase kinase 13
Details	13	97	hsa-miR-205-5p	NFAT5	nuclear factor of activated T cells 5
Details	14	97	hsa-miR-205-5p	C9orf153	chromosome 9 open reading frame 153
Details	15	97	hsa-miR-205-5p	TAPT1	transmembrane anterior posterior transformation 1
Details	16	97	hsa-miR-205-5p	CCNJ	cyclin J
Details	17	97	hsa-miR-205-5p	DSC2	desmocollin 2
Details	18	96	hsa-miR-205-5p	MGRN1	mahogunin ring finger 1
Details	19	96	hsa-miR-205-5p	ZFYVE16	zinc finger FYVE-type containing 16
Details	20	96	hsa-miR-205-5p	CPSF6	cleavage and polyadenylation specific factor 6
Details	21	96	hsa-miR-205-5p	PLCB1	phospholipase C beta 1
Details	22	96	hsa-miR-205-5p	ZNF606	zinc finger protein 606
Details	23	96	hsa-miR-205-5p	CALCRL	calcitonin receptor like receptor
Details	24	96	hsa-miR-205-5p	CADM1	cell adhesion molecule 1
Details	25	96	hsa-miR-205-5p	TNFAIP8	TNF alpha induced protein 8
Details	26	96	hsa-miR-205-5p	C11orf86	chromosome 11 open reading frame 86
Details	27	96	hsa-miR-205-5p	CDH11	cadherin 11
Details	28	95	hsa-miR-205-5p	SLC35B3	solute carrier family 35 member B3
Details	29	95	hsa-miR-205-5p	CASC4	cancer susceptibility 4
Details	30	95	hsa-miR-205-5p	PRKCE	protein kinase C epsilon
Details	31	95	hsa-miR-205-5p	HS3ST1	heparan sulfate-glucosamine 3-sulfotransferase 1
Details	32	95	hsa-miR-205-5p	NECAP1	NECAP endocytosis associated 1
Details	33	95	hsa-miR-205-5p	LRP6	LDL receptor related protein 6
Details	34	95	hsa-miR-205-5p	LCOR	ligand dependent nuclear receptor corepressor
Details	35	95	hsa-miR-205-5p	MAGI2	membrane associated guanylate kinase, WW and PDZ domain containing 2
Details	36	95	hsa-miR-205-5p	VTI1B	vesicle transport through interaction with t-SNAREs 1B

Details	37	94	hsa-miR-205-5p	PDE3B	phosphodiesterase 3B
Details	38	94	hsa-miR-205-5p	CDK14	cyclin dependent kinase 14
Details	39	94	hsa-miR-205-5p	ROCK2	Rho associated coiled-coil containing protein kinase 2
Details	40	94	hsa-miR-205-5p	CCDC80	coiled-coil domain containing 80
Details	41	94	hsa-miR-205-5p	PTPRM	protein tyrosine phosphatase, receptor type M
Details	42	94	hsa-miR-205-5p	QKI	QKI, KH domain containing RNA binding
Details	43	94	hsa-miR-205-5p	SPANXN5	SPANX family member N5
Details	44	94	hsa-miR-205-5p	CPEB2	cytoplasmic polyadenylation element binding protein 2
Details	45	94	hsa-miR-205-5p	AMOT	angiomotin
Details	46	94	hsa-miR-205-5p	SLC19A2	solute carrier family 19 member 2
Details	47	94	hsa-miR-205-5p	HERC3	HECT and RLD domain containing E3 ubiquitin protein ligase 3
Details	48	94	hsa-miR-205-5p	EVA1C	eva-1 homolog C
Details	49	94	hsa-miR-205-5p	ERBB4	erb-b2 receptor tyrosine kinase 4
Details	50	94	hsa-miR-205-5p	DUSP7	dual specificity phosphatase 7
Details	51	93	hsa-miR-205-5p	DMXL2	Dmx like 2
Details	52	93	hsa-miR-205-5p	NKD1	NKD1, WNT signaling pathway inhibitor
Details	53	93	hsa-miR-205-5p	AFDN	afadin, adherens junction formation factor
Details	54	93	hsa-miR-205-5p	NFIB	nuclear factor I B
Details	55	93	hsa-miR-205-5p	ERRFI1	ERBB receptor feedback inhibitor 1
Details	56	93	hsa-miR-205-5p	EZR	ezrin
Details	57	93	hsa-miR-205-5p	ETNK1	ethanolamine kinase 1
Details	58	93	hsa-miR-205-5p	STRBP	spermatid perinuclear RNA binding protein
Details	59	93	hsa-miR-205-5p	LRRK2	leucine rich repeat kinase 2
Details	60	93	hsa-miR-205-5p	GPC6	glypican 6
Details	61	93	hsa-miR-205-5p	RTN3	reticulon 3
Details	62	93	hsa-miR-205-5p	ANKRD50	ankyrin repeat domain 50
Details	63	93	hsa-miR-205-5p	FOXF1	forkhead box F1
Details	64	92	hsa-miR-205-5p	MSI2	musashi RNA binding protein 2
Details	65	92	hsa-miR-205-5p	CLTC	clathrin heavy chain
Details	66	92	hsa-miR-205-5p	HSF5	heat shock transcription factor 5
Details	67	92	hsa-miR-205-5p	SEPT4	septin 4
Details	68	92	hsa-miR-205-5p	SLC35A1	solute carrier family 35 member A1
Details	69	92	hsa-miR-205-5p	LRP1	LDL receptor related protein 1
Details	70	92	hsa-miR-205-5p	HSD17B11	hydroxysteroid 17-beta dehydrogenase 11
Details	71	92	hsa-miR-205-5p	SIPA1L1	signal induced proliferation associated 1 like 1
Details	72	92	hsa-miR-205-5p	INPP4A	inositol polyphosphate-4-phosphatase type I A
Details	73	92	hsa-miR-205-5p	P2RY1	purinergic receptor P2Y1
Details	74	92	hsa-miR-205-5p	GABRA4	gamma-aminobutyric acid type A receptor alpha4 subunit
Details	75	92	hsa-miR-205-5p	SATB2	SATB homeobox 2
Details	76	92	hsa-miR-205-5p	C6orf222	chromosome 6 open reading frame 222
Details	77	92	hsa-miR-205-5p	SGMS1	sphingomyelin synthase 1
Details	78	92	hsa-miR-205-5p	RORA	RAR related orphan receptor A
Details	79	92	hsa-miR-205-5p	MARCKS	myristoylated alanine rich protein kinase C substrate
Details	80	92	hsa-miR-205-5p	CLDN11	claudin 11
Details	81	91	hsa-miR-205-5p	PSD3	pleckstrin and Sec7 domain containing 3
Details	82	91	hsa-miR-205-5p	ZNF800	zinc finger protein 800
Details	83	91	hsa-miR-205-5p	RCBTB1	RCC1 and BTB domain containing protein 1

Details	84	91	hsa-miR-205-5p	ZEB1	zinc finger E-box binding homeobox 1
Details	85	91	hsa-miR-205-5p	E2F5	E2F transcription factor 5
Details	86	91	hsa-miR-205-5p	KPNA1	karyopherin subunit alpha 1
Details	87	91	hsa-miR-205-5p	ZNF652	zinc finger protein 652
Details	88	91	hsa-miR-205-5p	ERBB3	erb-b2 receptor tyrosine kinase 3
Details	89	91	hsa-miR-205-5p	ADAMTS9	ADAM metallopeptidase with thrombospondin type 1 motif 9
Details	90	91	hsa-miR-205-5p	LAMC1	laminin subunit gamma 1
Details	91	91	hsa-miR-205-5p	PAX9	paired box 9
Details	92	91	hsa-miR-205-5p	FZD3	frizzled class receptor 3
Details	93	90	hsa-miR-205-5p	KAT2B	lysine acetyltransferase 2B
Details	94	90	hsa-miR-205-5p	MAGI1	membrane associated guanylate kinase, WW and PDZ domain containing 1
Details	95	90	hsa-miR-205-5p	RUVBL1	RuvB like AAA ATPase 1
Details	96	90	hsa-miR-205-5p	TP53BP2	tumor protein p53 binding protein 2
Details	97	90	hsa-miR-205-5p	RBM41	RNA binding motif protein 41
Details	98	90	hsa-miR-205-5p	ABCD1	ATP binding cassette subfamily D member 1
Details	99	90	hsa-miR-205-5p	NACC2	NACC family member 2
Details	100	90	hsa-miR-205-5p	SORBS1	sorbin and SH3 domain containing 1
Details	101	90	hsa-miR-205-5p	ZNF655	zinc finger protein 655
Details	102	90	hsa-miR-205-5p	EPPK1	epiplakin 1
Details	103	90	hsa-miR-205-5p	LYSMD3	LysM domain containing 3
Details	104	90	hsa-miR-205-5p	NOTCH2	notch 2
Details	105	90	hsa-miR-205-5p	COX20	cytochrome c oxidase assembly factor COX20
Details	106	90	hsa-miR-205-5p	YAP1	Yes associated protein 1
Details	107	90	hsa-miR-205-5p	SECISBP2L	SECIS binding protein 2 like
Details	108	89	hsa-miR-205-5p	SBF2	SET binding factor 2
Details	109	89	hsa-miR-205-5p	AXIN2	axin 2
Details	110	89	hsa-miR-205-5p	SMAD4	SMAD family member 4
Details	111	89	hsa-miR-205-5p	VEGFA	vascular endothelial growth factor A
Details	112	89	hsa-miR-205-5p	PCNX1	pecanex 1
Details	113	89	hsa-miR-205-5p	JADE1	jade family PHD finger 1
Details	114	89	hsa-miR-205-5p	LHFPL2	LHFPL tetraspan subfamily member 2
Details	115	89	hsa-miR-205-5p	CDC27	cell division cycle 27
Details	116	89	hsa-miR-205-5p	TBC1D26	TBC1 domain family member 26
Details	117	89	hsa-miR-205-5p	CCNY	cyclin Y
Details	118	89	hsa-miR-205-5p	CMTM4	CKLF like MARVEL transmembrane domain containing 4
Details	119	89	hsa-miR-205-5p	SELENOT	selenoprotein T
Details	120	88	hsa-miR-205-5p	CSF1	colony stimulating factor 1
Details	121	88	hsa-miR-205-5p	KANSL3	KAT8 regulatory NSL complex subunit 3
Details	122	88	hsa-miR-205-5p	SPANXN1	SPANX family member N1
Details	123	88	hsa-miR-205-5p	EXOC6B	exocyst complex component 6B
Details	124	88	hsa-miR-205-5p	SNRPF	small nuclear ribonucleoprotein polypeptide F
Details	125	88	hsa-miR-205-5p	ALAD	aminolevulinate dehydratase
Details	126	88	hsa-miR-205-5p	TNPO1	transportin 1
Details	127	88	hsa-miR-205-5p	NAA30	N(alpha)-acetyltransferase 30, NatC catalytic subunit
Details	128	88	hsa-miR-205-5p	DSC1	desmocollin 1
Details	129	88	hsa-miR-205-5p	ZHX3	zinc fingers and homeoboxes 3
Details	130	88	hsa-miR-205-5p	NR3C2	nuclear receptor subfamily 3 group C member 2

Details	131	87	hsa-miR-205-5p	DCT	dopachrome tautomerase
Details	132	87	hsa-miR-205-5p	TRAK2	trafficking kinesin protein 2
Details	133	87	hsa-miR-205-5p	CALU	calumenin
Details	134	87	hsa-miR-205-5p	PPP1R15B	protein phosphatase 1 regulatory subunit 15B
Details	135	87	hsa-miR-205-5p	SMAD1	SMAD family member 1
Details	136	87	hsa-miR-205-5p	RUNX2	runt related transcription factor 2
Details	137	87	hsa-miR-205-5p	MTR	5-methyltetrahydrofolate-homocysteine methyltransferase
Details	138	87	hsa-miR-205-5p	SPRY1	sprouty RTK signaling antagonist 1
Details	139	87	hsa-miR-205-5p	SUSD1	sushi domain containing 1
Details	140	87	hsa-miR-205-5p	ENC1	ectodermal-neural cortex 1
Details	141	87	hsa-miR-205-5p	FAM122C	family with sequence similarity 122C
Details	142	87	hsa-miR-205-5p	SLC25A31	solute carrier family 25 member 31
Details	143	87	hsa-miR-205-5p	ZIK1	zinc finger protein interacting with K protein 1
Details	144	87	hsa-miR-205-5p	C12orf29	chromosome 12 open reading frame 29
Details	145	87	hsa-miR-205-5p	FRK	fyn related Src family tyrosine kinase
Details	146	86	hsa-miR-205-5p	DLG2	discs large MAGUK scaffold protein 2
Details	147	86	hsa-miR-205-5p	MTF1	metal regulatory transcription factor 1
Details	148	86	hsa-miR-205-5p	CXorf21	chromosome X open reading frame 21
Details	149	86	hsa-miR-205-5p	ATP5MC3	ATP synthase membrane subunit c locus 3
Details	150	86	hsa-miR-205-5p	HSPA13	heat shock protein family A (Hsp70) member 13
Details	151	86	hsa-miR-205-5p	FBXO22	F-box protein 22
Details	152	86	hsa-miR-205-5p	SH3GL3	SH3 domain containing GRB2 like 3, endophilin A3
Details	153	86	hsa-miR-205-5p	RBPMS2	RNA binding protein, mRNA processing factor 2
Details	154	86	hsa-miR-205-5p	MORF4L2	mortality factor 4 like 2
Details	155	86	hsa-miR-205-5p	SLC25A21	solute carrier family 25 member 21
Details	156	85	hsa-miR-205-5p	PJA2	praja ring finger ubiquitin ligase 2
Details	157	85	hsa-miR-205-5p	GXYLT1	glucoside xylosyltransferase 1
Details	158	85	hsa-miR-205-5p	SPINK13	serine peptidase inhibitor, Kazal type 13 (putative)
Details	159	85	hsa-miR-205-5p	COL4A1	collagen type IV alpha 1 chain
Details	160	85	hsa-miR-205-5p	ENPP4	ectonucleotide pyrophosphatase/phosphodiesterase 4
Details	161	85	hsa-miR-205-5p	NEU1	neuraminidase 1
Details	162	85	hsa-miR-205-5p	KDM4B	lysine demethylase 4B
Details	163	85	hsa-miR-205-5p	SCYL2	SCY1 like pseudokinase 2
Details	164	85	hsa-miR-205-5p	MINDY2	MINDY lysine 48 deubiquitinase 2
Details	165	85	hsa-miR-205-5p	LIN9	lin-9 DREAM MuvB core complex component
Details	166	85	hsa-miR-205-5p	MICAL2	microtubule associated monooxygenase, calponin and LIM domain containing 2
Details	167	85	hsa-miR-205-5p	PHYHIPL	phytanoyl-CoA 2-hydroxylase interacting protein like
Details	168	85	hsa-miR-205-5p	STK38L	serine/threonine kinase 38 like
Details	169	84	hsa-miR-205-5p	CLOCK	clock circadian regulator
Details	170	84	hsa-miR-205-5p	DHFR	dihydrofolate reductase
Details	171	84	hsa-miR-205-5p	RHPN2	rhophilin Rho GTPase binding protein 2
Details	172	84	hsa-miR-205-5p	KY	kyphoscoliosis peptidase
Details	173	84	hsa-miR-205-5p	TM9SF2	transmembrane 9 superfamily member 2
Details	174	84	hsa-miR-205-5p	AP1AR	adaptor related protein complex 1 associated regulatory protein

Details	175	84	hsa-miR-205-5p	ETFBKMT	electron transfer flavoprotein subunit beta lysine methyltransferase
Details	176	84	hsa-miR-205-5p	MTMR10	myotubularin related protein 10
Details	177	84	hsa-miR-205-5p	RNF4	ring finger protein 4
Details	178	83	hsa-miR-205-5p	AFF1	AF4/FMR2 family member 1
Details	179	83	hsa-miR-205-5p	SLC35A3	solute carrier family 35 member A3
Details	180	83	hsa-miR-205-5p	B4GALT5	beta-1,4-galactosyltransferase 5
Details	181	83	hsa-miR-205-5p	FAM114A2	family with sequence similarity 114 member A2
Details	182	83	hsa-miR-205-5p	MMD	monocyte to macrophage differentiation associated
Details	183	83	hsa-miR-205-5p	JADE3	jade family PHD finger 3
Details	184	83	hsa-miR-205-5p	NAA25	N(alpha)-acetyltransferase 25, NatB auxiliary subunit
Details	185	83	hsa-miR-205-5p	ZBTB20	zinc finger and BTB domain containing 20
Details	186	83	hsa-miR-205-5p	CFL2	cofilin 2
Details	187	83	hsa-miR-205-5p	EPB41	erythrocyte membrane protein band 4.1
Details	188	83	hsa-miR-205-5p	GPM6A	glycoprotein M6A
Details	189	82	hsa-miR-205-5p	EFNA2	ephrin A2
Details	190	82	hsa-miR-205-5p	AK4	adenylate kinase 4
Details	191	82	hsa-miR-205-5p	AP1G1	adaptor related protein complex 1 subunit gamma 1
Details	192	82	hsa-miR-205-5p	VAPA	VAMP associated protein A
Details	193	82	hsa-miR-205-5p	PTK7	protein tyrosine kinase 7 (inactive)
Details	194	82	hsa-miR-205-5p	INPPL1	inositol polyphosphate phosphatase like 1
Details	195	81	hsa-miR-205-5p	NIPSNAP3A	nipsnap homolog 3A
Details	196	81	hsa-miR-205-5p	ACSL1	acyl-CoA synthetase long chain family member 1
Details	197	81	hsa-miR-205-5p	AKAP11	A-kinase anchoring protein 11
Details	198	81	hsa-miR-205-5p	ADGRE2	adhesion G protein-coupled receptor E2
Details	199	81	hsa-miR-205-5p	FBXO33	F-box protein 33
Details	200	81	hsa-miR-205-5p	EEF1AKMT3	EEF1A lysine methyltransferase 3
Details	201	81	hsa-miR-205-5p	PROX1	prospero homeobox 1
Details	202	81	hsa-miR-205-5p	CCDC141	coiled-coil domain containing 141
Details	203	81	hsa-miR-205-5p	SERTAD2	SERTA domain containing 2
Details	204	81	hsa-miR-205-5p	MOB3B	MOB kinase activator 3B
Details	205	81	hsa-miR-205-5p	TOR1AIP2	torsin 1A interacting protein 2
Details	206	81	hsa-miR-205-5p	TPR	translocated promoter region, nuclear basket protein
Details	207	81	hsa-miR-205-5p	SLC4A4	solute carrier family 4 member 4
Details	208	81	hsa-miR-205-5p	CCT8L2	chaperonin containing TCP1 subunit 8 like 2
Details	209	81	hsa-miR-205-5p	KLK12	kallikrein related peptidase 12
Details	210	81	hsa-miR-205-5p	TBX3	T-box 3
Details	211	81	hsa-miR-205-5p	HSPA8	heat shock protein family A (Hsp70) member 8
Details	212	81	hsa-miR-205-5p	GLYATL1	glycine-N-acyltransferase like 1
Details	213	81	hsa-miR-205-5p	CAMSAP2	calmodulin regulated spectrin associated protein family member 2
Details	214	81	hsa-miR-205-5p	SLC30A8	solute carrier family 30 member 8
Details	215	81	hsa-miR-205-5p	RPS6KA3	ribosomal protein S6 kinase A3
Details	216	81	hsa-miR-205-5p	MGA	MGA, MAX dimerization protein
Details	217	81	hsa-miR-205-5p	NSF	N-ethylmaleimide sensitive factor, vesicle fusing ATPase

Details	218	80	hsa-miR-205-5p	CCM2	CCM2 scaffold protein
Details	219	80	hsa-miR-205-5p	ANKRD22	ankyrin repeat domain 22
Details	220	80	hsa-miR-205-5p	ITIH5	inter-alpha-trypsin inhibitor heavy chain family member 5
Details	221	80	hsa-miR-205-5p	PCP4L1	Purkinje cell protein 4 like 1
Details	222	80	hsa-miR-205-5p	GRAMD1C	GRAM domain containing 1C
Details	223	80	hsa-miR-205-5p	ESRRG	estrogen related receptor gamma
Details	224	80	hsa-miR-205-5p	E2F3	E2F transcription factor 3
Details	225	80	hsa-miR-205-5p	RAB3C	RAB3C, member RAS oncogene family
Details	226	80	hsa-miR-205-5p	SKAP2	src kinase associated phosphoprotein 2
Details	227	80	hsa-miR-205-5p	RAB14	RAB14, member RAS oncogene family
Details	228	80	hsa-miR-205-5p	PROX2	prospero homeobox 2
Details	229	80	hsa-miR-205-5p	SLC5A3	solute carrier family 5 member 3
Details	230	80	hsa-miR-205-5p	TAOK1	TAO kinase 1
Details	231	80	hsa-miR-205-5p	RAP2B	RAP2B, member of RAS oncogene family
Details	232	80	hsa-miR-205-5p	DISC1	DISC1 scaffold protein
Details	233	80	hsa-miR-205-5p	KMT2A	lysine methyltransferase 2A
Details	234	79	hsa-miR-205-5p	SERINC3	serine incorporator 3
Details	235	79	hsa-miR-205-5p	ZNF615	zinc finger protein 615
Details	236	79	hsa-miR-205-5p	ABHD2	abhydrolase domain containing 2
Details	237	79	hsa-miR-205-5p	DGCR8	DGCR8, microprocessor complex subunit
Details	238	79	hsa-miR-205-5p	MGAT4A	alpha-1,3-mannosyl-glycoprotein 4-beta-N-acetylglucosaminyltransferase A
Details	239	79	hsa-miR-205-5p	CASD1	CAS1 domain containing 1
Details	240	79	hsa-miR-205-5p	MTMR9	myotubularin related protein 9
Details	241	79	hsa-miR-205-5p	CFAP65	cilia and flagella associated protein 65
Details	242	79	hsa-miR-205-5p	CBX1	chromobox 1
Details	243	79	hsa-miR-205-5p	AREL1	apoptosis resistant E3 ubiquitin protein ligase 1
Details	244	79	hsa-miR-205-5p	TIMM17A	translocase of inner mitochondrial membrane 17A
Details	245	79	hsa-miR-205-5p	ABHD17B	abhydrolase domain containing 17B
Details	246	79	hsa-miR-205-5p	ASAH2B	N-acylsphingosine amidohydrolase 2B
Details	247	78	hsa-miR-205-5p	FAM155A	family with sequence similarity 155 member A
Details	248	78	hsa-miR-205-5p	SLC30A7	solute carrier family 30 member 7
Details	249	78	hsa-miR-205-5p	CREBRF	CREB3 regulatory factor
Details	250	78	hsa-miR-205-5p	SEMA7A	semaphorin 7A (John Milton Hagen blood group)
Details	251	78	hsa-miR-205-5p	VASN	vasorin
Details	252	78	hsa-miR-205-5p	TRIP12	thyroid hormone receptor interactor 12
Details	253	78	hsa-miR-205-5p	NCAM1	neural cell adhesion molecule 1
Details	254	78	hsa-miR-205-5p	MED1	mediator complex subunit 1
Details	255	78	hsa-miR-205-5p	NTNG1	netrin G1
Details	256	78	hsa-miR-205-5p	SHISA6	shisa family member 6
Details	257	78	hsa-miR-205-5p	DERL1	derlin 1
Details	258	78	hsa-miR-205-5p	B4GALT6	beta-1,4-galactosyltransferase 6
Details	259	77	hsa-miR-205-5p	BLOC1S5	biogenesis of lysosomal organelles complex 1 subunit 5
Details	260	77	hsa-miR-205-5p	SCMH1	Scm polycomb group protein homolog 1
Details	261	77	hsa-miR-205-5p	TGFA	transforming growth factor alpha
Details	262	77	hsa-miR-205-5p	COX15	cytochrome c oxidase assembly homolog COX15

Details	263	77	hsa-miR-205-5p	FMN1	formin 1
Details	264	77	hsa-miR-205-5p	PNN	pinin, desmosome associated protein
Details	265	77	hsa-miR-205-5p	PHC3	polyhomeotic homolog 3
Details	266	77	hsa-miR-205-5p	RNF180	ring finger protein 180
Details	267	77	hsa-miR-205-5p	ZCCHC14	zinc finger CCHC-type containing 14
Details	268	77	hsa-miR-205-5p	SIAH1	siah E3 ubiquitin protein ligase 1
Details	269	77	hsa-miR-205-5p	COX11	cytochrome c oxidase copper chaperone COX11
Details	270	77	hsa-miR-205-5p	ZMYM4	zinc finger MYM-type containing 4
Details	271	76	hsa-miR-205-5p	MDH2	malate dehydrogenase 2
Details	272	76	hsa-miR-205-5p	LPAR1	lysophosphatidic acid receptor 1
Details	273	76	hsa-miR-205-5p	SHLD2	shieldin complex subunit 2
Details	274	76	hsa-miR-205-5p	FAM104B	family with sequence similarity 104 member B
Details	275	76	hsa-miR-205-5p	UHRF1BP1L	UHRF1 binding protein 1 like
Details	276	76	hsa-miR-205-5p	CYBB	cytochrome b-245 beta chain
Details	277	76	hsa-miR-205-5p	CTPS2	CTP synthase 2
Details	278	76	hsa-miR-205-5p	ATP10A	ATPase phospholipid transporting 10A (putative)
Details	279	76	hsa-miR-205-5p	TASOR	transcription activation suppressor
Details	280	76	hsa-miR-205-5p	SCRN2	secernin 2
Details	281	75	hsa-miR-205-5p	NAA50	N(alpha)-acetyltransferase 50, NatE catalytic subunit
Details	282	75	hsa-miR-205-5p	SALL4	spalt like transcription factor 4
Details	283	75	hsa-miR-205-5p	ACTB	actin beta
Details	284	75	hsa-miR-205-5p	UGT3A1	UDP glycosyltransferase family 3 member A1
Details	285	75	hsa-miR-205-5p	YES1	YES proto-oncogene 1, Src family tyrosine kinase
Details	286	75	hsa-miR-205-5p	INSR	insulin receptor
Details	287	75	hsa-miR-205-5p	LTA	lymphotoxin alpha
Details	288	75	hsa-miR-205-5p	KLF12	Kruppel like factor 12
Details	289	75	hsa-miR-205-5p	SLC30A9	solute carrier family 30 member 9
Details	290	75	hsa-miR-205-5p	PHC2	polyhomeotic homolog 2
Details	291	75	hsa-miR-205-5p	INSYN2	inhibitory synaptic factor 2A
Details	292	74	hsa-miR-205-5p	HS3ST3A1	heparan sulfate-glucosamine 3-sulfotransferase 3A1
Details	293	74	hsa-miR-205-5p	EPHA4	EPH receptor A4
Details	294	74	hsa-miR-205-5p	IRF1	interferon regulatory factor 1
Details	295	74	hsa-miR-205-5p	CAMK4	calcium/calmodulin dependent protein kinase IV
Details	296	74	hsa-miR-205-5p	WWC1	WW and C2 domain containing 1
Details	297	74	hsa-miR-205-5p	IL1R1	interleukin 1 receptor type 1
Details	298	74	hsa-miR-205-5p	DRAM1	DNA damage regulated autophagy modulator 1
Details	299	74	hsa-miR-205-5p	ESM1	endothelial cell specific molecule 1
Details	300	74	hsa-miR-205-5p	MFSD14B	major facilitator superfamily domain containing 14B
Details	301	74	hsa-miR-205-5p	PEBP1	phosphatidylethanolamine binding protein 1
Details	302	74	hsa-miR-205-5p	MED13L	mediator complex subunit 13 like
Details	303	74	hsa-miR-205-5p	PAFAH1B1	platelet activating factor acetylhydrolase 1b regulatory subunit 1
Details	304	74	hsa-miR-205-5p	AVPR1A	arginine vasopressin receptor 1A
Details	305	74	hsa-miR-205-5p	LETM1	leucine zipper and EF-hand containing transmembrane protein 1

Details	306	74	hsa-miR-205-5p	CNNM1	cyclin and CBS domain divalent metal cation transport mediator 1
Details	307	74	hsa-miR-205-5p	PRPF38A	pre-mRNA processing factor 38A
Details	308	74	hsa-miR-205-5p	FAM84B	family with sequence similarity 84 member B
Details	309	74	hsa-miR-205-5p	AIG1	androgen induced 1
Details	310	74	hsa-miR-205-5p	TMED5	transmembrane p24 trafficking protein 5
Details	311	74	hsa-miR-205-5p	MSL2	MSL complex subunit 2
Details	312	74	hsa-miR-205-5p	BMPER	BMP binding endothelial regulator
Details	313	74	hsa-miR-205-5p	ZNFX1	zinc finger NFX1-type containing 1
Details	314	74	hsa-miR-205-5p	LINC02054	long intergenic non-protein coding RNA 2054
Details	315	73	hsa-miR-205-5p	OCIAD1	OCIA domain containing 1
Details	316	73	hsa-miR-205-5p	USP51	ubiquitin specific peptidase 51
Details	317	73	hsa-miR-205-5p	LMNA	lamin A/C
Details	318	73	hsa-miR-205-5p	ELMSAN1	ELM2 and Myb/SANT domain containing 1
Details	319	73	hsa-miR-205-5p	EDIL3	EGF like repeats and discoidin domains 3
Details	320	73	hsa-miR-205-5p	BMF	Bcl2 modifying factor
Details	321	73	hsa-miR-205-5p	KCNT2	potassium sodium-activated channel subfamily T member 2
Details	322	73	hsa-miR-205-5p	SHROOM3	shroom family member 3
Details	323	73	hsa-miR-205-5p	LYPD6	LY6/PLAUR domain containing 6
Details	324	73	hsa-miR-205-5p	INHBA	inhibin subunit beta A
Details	325	73	hsa-miR-205-5p	IRF2BPL	interferon regulatory factor 2 binding protein like
Details	326	73	hsa-miR-205-5p	PTP4A1	protein tyrosine phosphatase type IVA, member 1
Details	327	73	hsa-miR-205-5p	TNRC6B	trinucleotide repeat containing 6B
Details	328	73	hsa-miR-205-5p	CYTH3	cytohesin 3
Details	329	72	hsa-miR-205-5p	MFNG	MFNG O-fucosylpeptide 3-beta-N-acetylglucosaminyltransferase
Details	330	72	hsa-miR-205-5p	CHCHD3	coiled-coil-helix-coiled-coil-helix domain containing 3
Details	331	72	hsa-miR-205-5p	XBP1	X-box binding protein 1
Details	332	72	hsa-miR-205-5p	GALNT7	polypeptide N-acetylgalactosaminyltransferase 7
Details	333	72	hsa-miR-205-5p	PTCHD4	patched domain containing 4
Details	334	72	hsa-miR-205-5p	MIPOL1	mirror-image polydactyly 1
Details	335	72	hsa-miR-205-5p	APLNR	apelin receptor
Details	336	72	hsa-miR-205-5p	H2AFJ	H2A histone family member J
Details	337	72	hsa-miR-205-5p	BAMBI	BMP and activin membrane bound inhibitor
Details	338	72	hsa-miR-205-5p	TEAD1	TEA domain transcription factor 1
Details	339	71	hsa-miR-205-5p	HIPK3	homeodomain interacting protein kinase 3
Details	340	71	hsa-miR-205-5p	XPNPEP3	X-prolyl aminopeptidase 3
Details	341	71	hsa-miR-205-5p	KCNJ16	potassium voltage-gated channel subfamily J member 16
Details	342	71	hsa-miR-205-5p	THUMPD3	THUMP domain containing 3
Details	343	71	hsa-miR-205-5p	CHIC1	cysteine rich hydrophobic domain 1
Details	344	71	hsa-miR-205-5p	DYRK2	dual specificity tyrosine phosphorylation regulated kinase 2
Details	345	71	hsa-miR-205-5p	RAB23	RAB23, member RAS oncogene family
Details	346	71	hsa-miR-205-5p	LBHD1	LBH domain containing 1
Details	347	71	hsa-miR-205-5p	TRAF3	TNF receptor associated factor 3
Details	348	71	hsa-miR-205-5p	RALGAPB	Ral GTPase activating protein non-catalytic beta subunit

Details	349	71	hsa-miR-205-5p	ARL4D	ADP ribosylation factor like GTPase 4D
Details	350	71	hsa-miR-205-5p	PF4V1	platelet factor 4 variant 1
Details	351	70	hsa-miR-205-5p	TSC22D1	TSC22 domain family member 1
Details	352	70	hsa-miR-205-5p	TTC3	tetratricopeptide repeat domain 3
Details	353	70	hsa-miR-205-5p	TMEM144	transmembrane protein 144
Details	354	70	hsa-miR-205-5p	NIPA2	NIPA magnesium transporter 2
Details	355	70	hsa-miR-205-5p	JPH4	junctophilin 4
Details	356	70	hsa-miR-205-5p	CENPF	centromere protein F
Details	357	70	hsa-miR-205-5p	WDR48	WD repeat domain 48
Details	358	70	hsa-miR-205-5p	MSH2	mutS homolog 2
Details	359	70	hsa-miR-205-5p	WNK3	WNK lysine deficient protein kinase 3
Details	360	70	hsa-miR-205-5p	CASC1	cancer susceptibility 1
Details	361	70	hsa-miR-205-5p	AMER2	APC membrane recruitment protein 2
Details	362	70	hsa-miR-205-5p	NKX2-3	NK2 homeobox 3
Details	363	70	hsa-miR-205-5p	SEPT9	septin 9
Details	364	70	hsa-miR-205-5p	CRTC2	CREB regulated transcription coactivator 2
Details	365	69	hsa-miR-205-5p	BDP1	B double prime 1, subunit of RNA polymerase III transcription initiation factor IIIB
Details	366	69	hsa-miR-205-5p	C3orf80	chromosome 3 open reading frame 80
Details	367	69	hsa-miR-205-5p	CHMP2B	charged multivesicular body protein 2B
Details	368	69	hsa-miR-205-5p	PRKCA	protein kinase C alpha
Details	369	69	hsa-miR-205-5p	NEXN	nexilin F-actin binding protein
Details	370	69	hsa-miR-205-5p	DDX5	DEAD-box helicase 5
Details	371	69	hsa-miR-205-5p	C6orf89	chromosome 6 open reading frame 89
Details	372	69	hsa-miR-205-5p	LCA5	LCA5, lebercilin
Details	373	69	hsa-miR-205-5p	PHB	prohibitin
Details	374	69	hsa-miR-205-5p	UNC5C	unc-5 netrin receptor C
Details	375	69	hsa-miR-205-5p	B3GNT5	UDP-GlcNAc:betaGal beta-1,3-N-acetylglucosaminyltransferase 5
Details	376	69	hsa-miR-205-5p	ASB8	ankyrin repeat and SOCS box containing 8
Details	377	69	hsa-miR-205-5p	ZBTB18	zinc finger and BTB domain containing 18
Details	378	69	hsa-miR-205-5p	CHRAC1	chromatin accessibility complex subunit 1
Details	379	69	hsa-miR-205-5p	PIKFYVE	phosphoinositide kinase, FYVE-type zinc finger containing
Details	380	69	hsa-miR-205-5p	ZEB2	zinc finger E-box binding homeobox 2
Details	381	69	hsa-miR-205-5p	C3orf35	chromosome 3 open reading frame 35
Details	382	69	hsa-miR-205-5p	TM9SF3	transmembrane 9 superfamily member 3
Details	383	69	hsa-miR-205-5p	PATJ	PATJ, crumbs cell polarity complex component
Details	384	69	hsa-miR-205-5p	HCFC2	host cell factor C2
Details	385	68	hsa-miR-205-5p	STS	steroid sulfatase
Details	386	68	hsa-miR-205-5p	MYLK4	myosin light chain kinase family member 4
Details	387	68	hsa-miR-205-5p	HMGXB3	HMG-box containing 3
Details	388	68	hsa-miR-205-5p	STXBP6	syntaxin binding protein 6
Details	389	68	hsa-miR-205-5p	CARD8	caspase recruitment domain family member 8
Details	390	68	hsa-miR-205-5p	FXN	frataxin
Details	391	68	hsa-miR-205-5p	C10orf113	chromosome 10 open reading frame 113
Details	392	68	hsa-miR-205-5p	ZFP3	ZFP3 zinc finger protein
Details	393	67	hsa-miR-205-5p	PANK1	pantothenate kinase 1
Details	394	67	hsa-miR-205-5p	FRMD5	FERM domain containing 5
Details	395	67	hsa-miR-205-5p	SCNN1A	sodium channel epithelial 1 alpha subunit
Details	396	67	hsa-miR-205-5p	AP3M2	adaptor related protein complex 3 subunit mu 2

Details	397	67	hsa-miR-205-5p	DMXL1	Dmx like 1
Details	398	67	hsa-miR-205-5p	CWC27	CWC27 spliceosome associated protein homolog
Details	399	67	hsa-miR-205-5p	WWC2	WW and C2 domain containing 2
Details	400	67	hsa-miR-205-5p	FAM136A	family with sequence similarity 136 member A
Details	401	67	hsa-miR-205-5p	UBA52	ubiquitin A-52 residue ribosomal protein fusion product 1
Details	402	67	hsa-miR-205-5p	DNM1L	dynamin 1 like
Details	403	67	hsa-miR-205-5p	TBX21	T-box 21
Details	404	67	hsa-miR-205-5p	RELN	reelin
Details	405	67	hsa-miR-205-5p	GLIS3	GLIS family zinc finger 3
Details	406	66	hsa-miR-205-5p	MID1IP1	MID1 interacting protein 1
Details	407	66	hsa-miR-205-5p	CETN1	centrin 1
Details	408	66	hsa-miR-205-5p	HOOK3	hook microtubule tethering protein 3
Details	409	66	hsa-miR-205-5p	ABCD2	ATP binding cassette subfamily D member 2
Details	410	66	hsa-miR-205-5p	ABHD10	abhydrolase domain containing 10
Details	411	66	hsa-miR-205-5p	SLC6A15	solute carrier family 6 member 15
Details	412	66	hsa-miR-205-5p	KYAT3	kynurenine aminotransferase 3
Details	413	66	hsa-miR-205-5p	COBL	cordon-bleu WH2 repeat protein
Details	414	66	hsa-miR-205-5p	USP29	ubiquitin specific peptidase 29
Details	415	66	hsa-miR-205-5p	FBXO41	F-box protein 41
Details	416	66	hsa-miR-205-5p	TENT4B	terminal nucleotidyltransferase 4B
Details	417	66	hsa-miR-205-5p	CYLC2	cylicin 2
Details	418	66	hsa-miR-205-5p	GRAMD2A	GRAM domain containing 2A
Details	419	66	hsa-miR-205-5p	DDX52	DExD-box helicase 52
Details	420	66	hsa-miR-205-5p	ZNF208	zinc finger protein 208
Details	421	66	hsa-miR-205-5p	STAG1	stromal antigen 1
Details	422	66	hsa-miR-205-5p	LIMS2	LIM zinc finger domain containing 2
Details	423	66	hsa-miR-205-5p	SP4	Sp4 transcription factor
Details	424	66	hsa-miR-205-5p	ABI2	abl interactor 2
Details	425	65	hsa-miR-205-5p	TCAIM	T cell activation inhibitor, mitochondrial
Details	426	65	hsa-miR-205-5p	GARS	glycyl-tRNA synthetase
Details	427	65	hsa-miR-205-5p	GATA3	GATA binding protein 3
Details	428	65	hsa-miR-205-5p	TP53INP1	tumor protein p53 inducible nuclear protein 1
Details	429	65	hsa-miR-205-5p	TTC19	tetratricopeptide repeat domain 19
Details	430	65	hsa-miR-205-5p	ZBTB33	zinc finger and BTB domain containing 33
Details	431	65	hsa-miR-205-5p	TRA2A	transformer 2 alpha homolog
Details	432	65	hsa-miR-205-5p	SEPT5	septin 5
Details	433	65	hsa-miR-205-5p	HOXD9	homeobox D9
Details	434	65	hsa-miR-205-5p	PSMA4	proteasome subunit alpha 4
Details	435	65	hsa-miR-205-5p	CNIH1	cornichon family AMPA receptor auxiliary protein 1
Details	436	64	hsa-miR-205-5p	KDELR3	KDEL endoplasmic reticulum protein retention receptor 3
Details	437	64	hsa-miR-205-5p	XPO4	exportin 4
Details	438	64	hsa-miR-205-5p	IKZF3	IKAROS family zinc finger 3
Details	439	64	hsa-miR-205-5p	CDH7	cadherin 7
Details	440	64	hsa-miR-205-5p	CNTN5	contactin 5
Details	441	64	hsa-miR-205-5p	PTEN	phosphatase and tensin homolog
Details	442	64	hsa-miR-205-5p	TXNRD1	thioredoxin reductase 1
Details	443	64	hsa-miR-205-5p	WWC3	WWC family member 3

Details	444	64	hsa-miR-205-5p	PICALM	phosphatidylinositol binding clathrin assembly protein
Details	445	64	hsa-miR-205-5p	GCC2	GRIP and coiled-coil domain containing 2
Details	446	64	hsa-miR-205-5p	STK3	serine/threonine kinase 3
Details	447	64	hsa-miR-205-5p	INO80D	INO80 complex subunit D
Details	448	64	hsa-miR-205-5p	PKD2L1	polycystin 2 like 1, transient receptor potential cation channel
Details	449	64	hsa-miR-205-5p	RGPD5	RANBP2-like and GRIP domain containing 5
Details	450	64	hsa-miR-205-5p	IL6ST	interleukin 6 signal transducer
Details	451	64	hsa-miR-205-5p	NHLRC2	NHL repeat containing 2
Details	452	64	hsa-miR-205-5p	SULF1	sulfatase 1
Details	453	64	hsa-miR-205-5p	NDUFA4	NDUFA4, mitochondrial complex associated
Details	454	64	hsa-miR-205-5p	LIN54	lin-54 DREAM MuvB core complex component
Details	455	63	hsa-miR-205-5p	SMAD2	SMAD family member 2
Details	456	63	hsa-miR-205-5p	PGAP3	post-GPI attachment to proteins 3
Details	457	63	hsa-miR-205-5p	GREM2	gremlin 2, DAN family BMP antagonist
Details	458	63	hsa-miR-205-5p	PTGFR	prostaglandin F receptor
Details	459	63	hsa-miR-205-5p	ELF1	E74 like ETS transcription factor 1
Details	460	63	hsa-miR-205-5p	PUM1	pumilio RNA binding family member 1
Details	461	63	hsa-miR-205-5p	RGPD4	RANBP2-like and GRIP domain containing 4
Details	462	63	hsa-miR-205-5p	RUBCN	rubicon autophagy regulator
Details	463	63	hsa-miR-205-5p	GNAQ	G protein subunit alpha q
Details	464	63	hsa-miR-205-5p	RGPD8	RANBP2-like and GRIP domain containing 8
Details	465	63	hsa-miR-205-5p	PHETA2	PH domain containing endocytic trafficking adaptor 2
Details	466	63	hsa-miR-205-5p	C1orf229	chromosome 1 open reading frame 229
Details	467	63	hsa-miR-205-5p	RGPD6	RANBP2-like and GRIP domain containing 6
Details	468	63	hsa-miR-205-5p	AARS2	alanyl-tRNA synthetase 2, mitochondrial
Details	469	63	hsa-miR-205-5p	SEPT11	septin 11
Details	470	63	hsa-miR-205-5p	ARL13B	ADP ribosylation factor like GTPase 13B
Details	471	63	hsa-miR-205-5p	USP13	ubiquitin specific peptidase 13
Details	472	63	hsa-miR-205-5p	DCAF5	DDB1 and CUL4 associated factor 5
Details	473	63	hsa-miR-205-5p	TFDP2	transcription factor Dp-2
Details	474	62	hsa-miR-205-5p	CC2D2B	coiled-coil and C2 domain containing 2B
Details	475	62	hsa-miR-205-5p	LDLRAD3	low density lipoprotein receptor class A domain containing 3
Details	476	62	hsa-miR-205-5p	ZNF701	zinc finger protein 701
Details	477	62	hsa-miR-205-5p	GRB14	growth factor receptor bound protein 14
Details	478	62	hsa-miR-205-5p	NIPSNAP3B	nipsnap homolog 3B
Details	479	62	hsa-miR-205-5p	USP38	ubiquitin specific peptidase 38
Details	480	62	hsa-miR-205-5p	MDM4	MDM4, p53 regulator
Details	481	62	hsa-miR-205-5p	UNC5D	unc-5 netrin receptor D
Details	482	62	hsa-miR-205-5p	CREB1	cAMP responsive element binding protein 1
Details	483	62	hsa-miR-205-5p	COPS7B	COP9 signalosome subunit 7B
Details	484	62	hsa-miR-205-5p	CDIP1	cell death inducing p53 target 1
Details	485	62	hsa-miR-205-5p	GAB2	GRB2 associated binding protein 2
Details	486	62	hsa-miR-205-5p	RNGTT	RNA guanylyltransferase and 5'-phosphatase
Details	487	62	hsa-miR-205-5p	ZNF124	zinc finger protein 124
Details	488	61	hsa-miR-205-5p	PDK3	pyruvate dehydrogenase kinase 3
Details	489	61	hsa-miR-205-5p	PRMT2	protein arginine methyltransferase 2
Details	490	61	hsa-miR-205-5p	CPT1A	carnitine palmitoyltransferase 1A
Details	491	61	hsa-miR-205-5p	ZSWIM4	zinc finger SWIM-type containing 4

Details	492	61	hsa-miR-205-5p	SNX27	sorting nexin family member 27
Details	493	61	hsa-miR-205-5p	BORCS7	BLOC-1 related complex subunit 7
Details	494	61	hsa-miR-205-5p	DCAF1	DDB1 and CUL4 associated factor 1
Details	495	61	hsa-miR-205-5p	CUTC	cutC copper transporter
Details	496	61	hsa-miR-205-5p	LONRF2	LON peptidase N-terminal domain and ring finger 2
Details	497	61	hsa-miR-205-5p	KIF26B	kinesin family member 26B
Details	498	61	hsa-miR-205-5p	CD302	CD302 molecule
Details	499	61	hsa-miR-205-5p	PARD6B	par-6 family cell polarity regulator beta
Details	500	61	hsa-miR-205-5p	GSE1	Gse1 coiled-coil protein
Details	501	61	hsa-miR-205-5p	GJB7	gap junction protein beta 7
Details	502	61	hsa-miR-205-5p	ELK3	ELK3, ETS transcription factor
Details	503	61	hsa-miR-205-5p	B4GALT4	beta-1,4-galactosyltransferase 4
Details	504	61	hsa-miR-205-5p	LY75-CD302	LY75-CD302 readthrough
Details	505	60	hsa-miR-205-5p	ACVR2A	activin A receptor type 2A
Details	506	60	hsa-miR-205-5p	C5orf22	chromosome 5 open reading frame 22
Details	507	60	hsa-miR-205-5p	CEP126	centrosomal protein 126
Details	508	60	hsa-miR-205-5p	SMIM14	small integral membrane protein 14
Details	509	60	hsa-miR-205-5p	PPP1R3A	protein phosphatase 1 regulatory subunit 3A
Details	510	60	hsa-miR-205-5p	NEGR1	neuronal growth regulator 1
Details	511	60	hsa-miR-205-5p	SAMD5	sterile alpha motif domain containing 5
Details	512	60	hsa-miR-205-5p	RPS6KA5	ribosomal protein S6 kinase A5
Details	513	60	hsa-miR-205-5p	DDX55	DEAD-box helicase 55
Details	514	60	hsa-miR-205-5p	TMEM267	transmembrane protein 267
Details	515	60	hsa-miR-205-5p	SIRT5	sirtuin 5
Details	516	60	hsa-miR-205-5p	FUT9	fucosyltransferase 9
Details	517	60	hsa-miR-205-5p	NCAM2	neural cell adhesion molecule 2
Details	518	60	hsa-miR-205-5p	ZBTB2	zinc finger and BTB domain containing 2
Details	519	60	hsa-miR-205-5p	CCDC43	coiled-coil domain containing 43
Details	520	60	hsa-miR-205-5p	STYK1	serine/threonine/tyrosine kinase 1
Details	521	60	hsa-miR-205-5p	TMEM74	transmembrane protein 74
Details	522	60	hsa-miR-205-5p	TNFSF4	TNF superfamily member 4
Details	523	60	hsa-miR-205-5p	PCSK5	proprotein convertase subtilisin/kexin type 5
Details	524	60	hsa-miR-205-5p	EMX2	empty spiracles homeobox 2
Details	525	60	hsa-miR-205-5p	FYTTD1	forty-two-three domain containing 1
Details	526	60	hsa-miR-205-5p	SQLE	squalene epoxidase
Details	527	60	hsa-miR-205-5p	SMOC1	SPARC related modular calcium binding 1
Details	528	60	hsa-miR-205-5p	TMEM245	transmembrane protein 245
Details	529	60	hsa-miR-205-5p	MYO5B	myosin VB
Details	530	60	hsa-miR-205-5p	POC1B	POC1 centriolar protein B
Details	531	59	hsa-miR-205-5p	FAAP24	FA core complex associated protein 24
Details	532	59	hsa-miR-205-5p	CD163L1	CD163 molecule like 1
Details	533	59	hsa-miR-205-5p	TRDMT1	tRNA aspartic acid methyltransferase 1
Details	534	59	hsa-miR-205-5p	STMN4	stathmin 4
Details	535	59	hsa-miR-205-5p	ATP6V0A4	ATPase H+ transporting V0 subunit a4
Details	536	59	hsa-miR-205-5p	NUDT16	nudix hydrolase 16
Details	537	59	hsa-miR-205-5p	AEBP2	AE binding protein 2
Details	538	59	hsa-miR-205-5p	FRAT1	FRAT1, WNT signaling pathway regulator
Details	539	59	hsa-miR-205-5p	DUSP5	dual specificity phosphatase 5
Details	540	59	hsa-miR-205-5p	VIP	vasoactive intestinal peptide
Details	541	59	hsa-miR-205-5p	MECR	mitochondrial trans-2-enoyl-CoA reductase

Details	542	59	hsa-miR-205-5p	BMPR1B	bone morphogenetic protein receptor type 1B
Details	543	59	hsa-miR-205-5p	NCOA4	nuclear receptor coactivator 4
Details	544	59	hsa-miR-205-5p	CD24	CD24 molecule
Details	545	59	hsa-miR-205-5p	PPM1L	protein phosphatase, Mg2+/Mn2+ dependent 1L
Details	546	59	hsa-miR-205-5p	SACS	sacsin molecular chaperone
Details	547	59	hsa-miR-205-5p	GPAT4	glycerol-3-phosphate acyltransferase 4
Details	548	59	hsa-miR-205-5p	ACSL5	acyl-CoA synthetase long chain family member 5
Details	549	58	hsa-miR-205-5p	ERI1	exoribonuclease 1
Details	550	58	hsa-miR-205-5p	CCDC121	coiled-coil domain containing 121
Details	551	58	hsa-miR-205-5p	RARA	retinoic acid receptor alpha
Details	552	58	hsa-miR-205-5p	ENY2	ENY2, transcription and export complex 2 subunit
Details	553	58	hsa-miR-205-5p	ZDHHC9	zinc finger DHHC-type containing 9
Details	554	58	hsa-miR-205-5p	GLRB	glycine receptor beta
Details	555	58	hsa-miR-205-5p	ZNF184	zinc finger protein 184
Details	556	58	hsa-miR-205-5p	SEMA4C	semaphorin 4C
Details	557	58	hsa-miR-205-5p	CXorf40B	chromosome X open reading frame 40B
Details	558	58	hsa-miR-205-5p	GIGYF2	GRB10 interacting GYF protein 2
Details	559	58	hsa-miR-205-5p	TBX18	T-box 18
Details	560	58	hsa-miR-205-5p	CENPO	centromere protein O
Details	561	58	hsa-miR-205-5p	C10orf53	chromosome 10 open reading frame 53
Details	562	58	hsa-miR-205-5p	AGBL3	ATP/GTP binding protein like 3
Details	563	58	hsa-miR-205-5p	PPP1R8	protein phosphatase 1 regulatory subunit 8
Details	564	58	hsa-miR-205-5p	KIAA1324L	KIAA1324 like
Details	565	58	hsa-miR-205-5p	RNF165	ring finger protein 165
Details	566	58	hsa-miR-205-5p	CIAO1	cytosolic iron-sulfur assembly component 1
Details	567	57	hsa-miR-205-5p	ITGB6	integrin subunit beta 6
Details	568	57	hsa-miR-205-5p	MTERF1	mitochondrial transcription termination factor 1
Details	569	57	hsa-miR-205-5p	TTC9C	tetratricopeptide repeat domain 9C
Details	570	57	hsa-miR-205-5p	RNF144A	ring finger protein 144A
Details	571	57	hsa-miR-205-5p	CSRNP3	cysteine and serine rich nuclear protein 3
Details	572	57	hsa-miR-205-5p	TIPARP	TCDD inducible poly(ADP-ribose) polymerase
Details	573	57	hsa-miR-205-5p	ZFP91	ZFP91 zinc finger protein
Details	574	57	hsa-miR-205-5p	CUX2	cut like homeobox 2
Details	575	57	hsa-miR-205-5p	RAD17	RAD17 checkpoint clamp loader component
Details	576	57	hsa-miR-205-5p	DRAM2	DNA damage regulated autophagy modulator 2
Details	577	57	hsa-miR-205-5p	POU2F1	POU class 2 homeobox 1
Details	578	57	hsa-miR-205-5p	PLEK	pleckstrin
Details	579	57	hsa-miR-205-5p	EPHX4	epoxide hydrolase 4
Details	580	57	hsa-miR-205-5p	C10orf25	chromosome 10 open reading frame 25
Details	581	57	hsa-miR-205-5p	ZBTB34	zinc finger and BTB domain containing 34
Details	582	57	hsa-miR-205-5p	NFIA	nuclear factor I A
Details	583	57	hsa-miR-205-5p	DNHD1	dynein heavy chain domain 1
Details	584	57	hsa-miR-205-5p	DYRK1A	dual specificity tyrosine phosphorylation regulated kinase 1A
Details	585	57	hsa-miR-205-5p	MAP6	microtubule associated protein 6
Details	586	57	hsa-miR-205-5p	ELAVL4	ELAV like RNA binding protein 4

Details	587	57	hsa-miR-205-5p	FSD1L	fibronectin type III and SPRY domain containing 1 like
Details	588	56	hsa-miR-205-5p	FUT1	fucosyltransferase 1 (H blood group)
Details	589	56	hsa-miR-205-5p	C17orf97	chromosome 17 open reading frame 97
Details	590	56	hsa-miR-205-5p	CBLL1	Cbl proto-oncogene like 1
Details	591	56	hsa-miR-205-5p	AFF2	AF4/FMR2 family member 2
Details	592	56	hsa-miR-205-5p	CDC42BPB	CDC42 binding protein kinase beta
Details	593	56	hsa-miR-205-5p	ANGPT2	angiopoietin 2
Details	594	56	hsa-miR-205-5p	LHFPL4	LHFPL tetraspan subfamily member 4
Details	595	56	hsa-miR-205-5p	DPP10	dipeptidyl peptidase like 10
Details	596	56	hsa-miR-205-5p	PPP1R1C	protein phosphatase 1 regulatory inhibitor subunit 1C
Details	597	56	hsa-miR-205-5p	STK38	serine/threonine kinase 38
Details	598	56	hsa-miR-205-5p	TLE4	TLE family member 4, transcriptional corepressor
Details	599	56	hsa-miR-205-5p	RND3	Rho family GTPase 3
Details	600	56	hsa-miR-205-5p	WDTC1	WD and tetratricopeptide repeats 1
Details	601	56	hsa-miR-205-5p	CLDN8	claudin 8
Details	602	56	hsa-miR-205-5p	VEZF1	vascular endothelial zinc finger 1
Details	603	56	hsa-miR-205-5p	RABEP1	rabaptin, RAB GTPase binding effector protein 1
Details	604	55	hsa-miR-205-5p	TET1	tet methylcytosine dioxygenase 1
Details	605	55	hsa-miR-205-5p	N4BP2	NEDD4 binding protein 2
Details	606	55	hsa-miR-205-5p	MLPH	melanophilin
Details	607	55	hsa-miR-205-5p	DGKG	diacylglycerol kinase gamma
Details	608	55	hsa-miR-205-5p	SAMD4A	sterile alpha motif domain containing 4A
Details	609	55	hsa-miR-205-5p	E2F1	E2F transcription factor 1
Details	610	55	hsa-miR-205-5p	LPGAT1	lysophosphatidylglycerol acyltransferase 1
Details	611	55	hsa-miR-205-5p	IBA57	IBA57, iron-sulfur cluster assembly
Details	612	55	hsa-miR-205-5p	CAB39	calcium binding protein 39
Details	613	55	hsa-miR-205-5p	NEK6	NIMA related kinase 6
Details	614	55	hsa-miR-205-5p	KLHL2	kelch like family member 2
Details	615	55	hsa-miR-205-5p	TGOLN2	trans-golgi network protein 2
Details	616	55	hsa-miR-205-5p	MPRIP	myosin phosphatase Rho interacting protein
Details	617	55	hsa-miR-205-5p	DNAJC15	DnaJ heat shock protein family (Hsp40) member C15
Details	618	55	hsa-miR-205-5p	ERCC4	ERCC excision repair 4, endonuclease catalytic subunit
Details	619	55	hsa-miR-205-5p	OGFRL1	opioid growth factor receptor like 1
Details	620	55	hsa-miR-205-5p	NAV1	neuron navigator 1
Details	621	55	hsa-miR-205-5p	RAB9B	RAB9B, member RAS oncogene family
Details	622	55	hsa-miR-205-5p	TC2N	tandem C2 domains, nuclear
Details	623	55	hsa-miR-205-5p	WDR35	WD repeat domain 35
Details	624	55	hsa-miR-205-5p	EIF2S1	eukaryotic translation initiation factor 2 subunit alpha
Details	625	55	hsa-miR-205-5p	BRINP3	BMP/retinoic acid inducible neural specific 3
Details	626	55	hsa-miR-205-5p	C5orf47	chromosome 5 open reading frame 47
Details	627	54	hsa-miR-205-5p	SYT13	synaptotagmin 13
Details	628	54	hsa-miR-205-5p	KBTBD8	kelch repeat and BTB domain containing 8
Details	629	54	hsa-miR-205-5p	RAB11FIP4	RAB11 family interacting protein 4
Details	630	54	hsa-miR-205-5p	SRSF10	serine and arginine rich splicing factor 10
Details	631	54	hsa-miR-205-5p	STON2	stonin 2

Details	632	54	hsa-miR-205-5p	UCHL3	ubiquitin C-terminal hydrolase L3
Details	633	54	hsa-miR-205-5p	PPM1E	protein phosphatase, Mg2+/Mn2+ dependent 1E
Details	634	54	hsa-miR-205-5p	SLC7A6	solute carrier family 7 member 6
Details	635	54	hsa-miR-205-5p	EYA3	EYA transcriptional coactivator and phosphatase 3
Details	636	54	hsa-miR-205-5p	TMEM14B	transmembrane protein 14B
Details	637	54	hsa-miR-205-5p	NCAN	neurocan
Details	638	54	hsa-miR-205-5p	SV2B	synaptic vesicle glycoprotein 2B
Details	639	54	hsa-miR-205-5p	GMFB	glia maturation factor beta
Details	640	54	hsa-miR-205-5p	WFDC6	WAP four-disulfide core domain 6
Details	641	54	hsa-miR-205-5p	DOK1	docking protein 1
Details	642	54	hsa-miR-205-5p	FERMT2	fermitin family member 2
Details	643	54	hsa-miR-205-5p	C5orf64	chromosome 5 open reading frame 64
Details	644	54	hsa-miR-205-5p	ATG5	autophagy related 5
Details	645	53	hsa-miR-205-5p	PTP4A2	protein tyrosine phosphatase type IVA, member 2
Details	646	53	hsa-miR-205-5p	RAPGEF4	Rap guanine nucleotide exchange factor 4
Details	647	53	hsa-miR-205-5p	PRDM12	PR/SET domain 12
Details	648	53	hsa-miR-205-5p	RBBP4	RB binding protein 4, chromatin remodeling factor
Details	649	53	hsa-miR-205-5p	EIF4E	eukaryotic translation initiation factor 4E
Details	650	53	hsa-miR-205-5p	CHD2	chromodomain helicase DNA binding protein 2
Details	651	53	hsa-miR-205-5p	SSR1	signal sequence receptor subunit 1
Details	652	53	hsa-miR-205-5p	PSD4	pleckstrin and Sec7 domain containing 4
Details	653	53	hsa-miR-205-5p	KIF13B	kinesin family member 13B
Details	654	53	hsa-miR-205-5p	THBS1	thrombospondin 1
Details	655	53	hsa-miR-205-5p	ZC3H11A	zinc finger CCCH-type containing 11A
Details	656	53	hsa-miR-205-5p	CDRT1	CMT1A duplicated region transcript 1
Details	657	53	hsa-miR-205-5p	SEH1L	SEH1 like nucleoporin
Details	658	53	hsa-miR-205-5p	ALDH3B1	aldehyde dehydrogenase 3 family member B1
Details	659	53	hsa-miR-205-5p	FAM218A	family with sequence similarity 218 member A
Details	660	53	hsa-miR-205-5p	KIF5C	kinesin family member 5C
Details	661	53	hsa-miR-205-5p	CD99	CD99 molecule (Xg blood group)
Details	662	53	hsa-miR-205-5p	POTEM	POTE ankyrin domain family member M
Details	663	53	hsa-miR-205-5p	LILRB2	leukocyte immunoglobulin like receptor B2
Details	664	53	hsa-miR-205-5p	RNF157	ring finger protein 157
Details	665	53	hsa-miR-205-5p	UFD1	ubiquitin recognition factor in ER associated degradation 1
Details	666	53	hsa-miR-205-5p	CEACAM6	carcinoembryonic antigen related cell adhesion molecule 6
Details	667	52	hsa-miR-205-5p	GP6	glycoprotein VI platelet
Details	668	52	hsa-miR-205-5p	EXOG	exo/endonuclease G
Details	669	52	hsa-miR-205-5p	TRMT1L	tRNA methyltransferase 1 like
Details	670	52	hsa-miR-205-5p	GRIA4	glutamate ionotropic receptor AMPA type subunit 4
Details	671	52	hsa-miR-205-5p	CDPF1	cysteine rich DPF motif domain containing 1
Details	672	52	hsa-miR-205-5p	CXorf40A	chromosome X open reading frame 40A
Details	673	52	hsa-miR-205-5p	ADAM28	ADAM metallopeptidase domain 28
Details	674	52	hsa-miR-205-5p	SIDT1	SID1 transmembrane family member 1
Details	675	52	hsa-miR-205-5p	IQGAP1	IQ motif containing GTPase activating protein 1

Details	676	52	hsa-miR-205-5p	EPB41L1	erythrocyte membrane protein band 4.1 like 1
Details	677	52	hsa-miR-205-5p	TRIAP1	TP53 regulated inhibitor of apoptosis 1
Details	678	52	hsa-miR-205-5p	CHST6	carbohydrate sulfotransferase 6
Details	679	52	hsa-miR-205-5p	CLIP1	CAP-Gly domain containing linker protein 1
Details	680	52	hsa-miR-205-5p	TEX35	testis expressed 35
Details	681	52	hsa-miR-205-5p	SALL2	spalt like transcription factor 2
Details	682	52	hsa-miR-205-5p	CALHM4	calcium homeostasis modulator family member 4
Details	683	52	hsa-miR-205-5p	GGA2	golgi associated, gamma adaptin ear containing, ARF binding protein 2
Details	684	52	hsa-miR-205-5p	RNF212B	ring finger protein 212B
Details	685	52	hsa-miR-205-5p	SH2D4B	SH2 domain containing 4B
Details	686	52	hsa-miR-205-5p	KALRN	kalirin RhoGEF kinase
Details	687	52	hsa-miR-205-5p	AGPAT4	1-acylglycerol-3-phosphate O-acyltransferase 4
Details	688	52	hsa-miR-205-5p	CFAP44	cilia and flagella associated protein 44
Details	689	52	hsa-miR-205-5p	MTRF1L	mitochondrial translational release factor 1 like
Details	690	52	hsa-miR-205-5p	ZNF518B	zinc finger protein 518B
Details	691	52	hsa-miR-205-5p	DACH2	dachshund family transcription factor 2
Details	692	52	hsa-miR-205-5p	HNRNPH3	heterogeneous nuclear ribonucleoprotein H3
Details	693	52	hsa-miR-205-5p	GABPA	GA binding protein transcription factor subunit alpha
Details	694	52	hsa-miR-205-5p	ZHX1	zinc fingers and homeoboxes 1
Details	695	52	hsa-miR-205-5p	NCALD	neurocalcin delta
Details	696	52	hsa-miR-205-5p	TSHR	thyroid stimulating hormone receptor
Details	697	52	hsa-miR-205-5p	MYOCD	myocardin
Details	698	52	hsa-miR-205-5p	ATP7A	ATPase copper transporting alpha
Details	699	52	hsa-miR-205-5p	STRIP2	striatin interacting protein 2
Details	700	52	hsa-miR-205-5p	TMEM170A	transmembrane protein 170A
Details	701	52	hsa-miR-205-5p	KIAA0319	KIAA0319
Details	702	51	hsa-miR-205-5p	EDN3	endothelin 3
Details	703	51	hsa-miR-205-5p	PDE7A	phosphodiesterase 7A
Details	704	51	hsa-miR-205-5p	PI15	peptidase inhibitor 15
Details	705	51	hsa-miR-205-5p	MBNL3	muscleblind like splicing regulator 3
Details	706	51	hsa-miR-205-5p	PNPLA8	patatin like phospholipase domain containing 8
Details	707	51	hsa-miR-205-5p	ERP29	endoplasmic reticulum protein 29
Details	708	51	hsa-miR-205-5p	ZXDC	ZXD family zinc finger C
Details	709	51	hsa-miR-205-5p	MGLL	monoglyceride lipase
Details	710	51	hsa-miR-205-5p	PTPN21	protein tyrosine phosphatase, non-receptor type 21
Details	711	51	hsa-miR-205-5p	RSPO3	R-spondin 3
Details	712	51	hsa-miR-205-5p	RAD54L2	RAD54 like 2
Details	713	51	hsa-miR-205-5p	EREG	epiregulin
Details	714	51	hsa-miR-205-5p	NR3C1	nuclear receptor subfamily 3 group C member 1
Details	715	51	hsa-miR-205-5p	PPP3R1	protein phosphatase 3 regulatory subunit B, alpha
Details	716	51	hsa-miR-205-5p	CYP4V2	cytochrome P450 family 4 subfamily V member 2
Details	717	51	hsa-miR-205-5p	HAL	histidine ammonia-lyase
Details	718	51	hsa-miR-205-5p	RASSF3	Ras association domain family member 3

Details	719	51	hsa-miR-205-5p	UBE2K	ubiquitin conjugating enzyme E2 K
Details	720	50	hsa-miR-205-5p	ZNF302	zinc finger protein 302
Details	721	50	hsa-miR-205-5p	AVL9	AVL9 cell migration associated
Details	722	50	hsa-miR-205-5p	BEAN1	brain expressed associated with NEDD4 1
Details	723	50	hsa-miR-205-5p	DIO2	iodothyronine deiodinase 2
Details	724	50	hsa-miR-205-5p	PTCHD1	patched domain containing 1
Details	725	50	hsa-miR-205-5p	NCAPG	non-SMC condensin I complex subunit G
Details	726	50	hsa-miR-205-5p	CAT	catalase
Details	727	50	hsa-miR-205-5p	CA8	carbonic anhydrase 8
Details	728	50	hsa-miR-205-5p	TCF20	transcription factor 20
Details	729	50	hsa-miR-205-5p	TOX	thymocyte selection associated high mobility group box
Details	730	50	hsa-miR-205-5p	GPD2	glycerol-3-phosphate dehydrogenase 2
Details	731	50	hsa-miR-205-5p	ARHGAP24	Rho GTPase activating protein 24
Details	732	50	hsa-miR-205-5p	RASL11B	RAS like family 11 member B
Details	733	50	hsa-miR-205-5p	STEAP2	STEAP2 metalloreductase
Details	734	50	hsa-miR-205-5p	GTF2H5	general transcription factor IIH subunit 5
Details	735	50	hsa-miR-205-5p	CDON	cell adhesion associated, oncogene regulated
Details	736	50	hsa-miR-205-5p	HACD4	3-hydroxyacyl-CoA dehydratase 4
Details	737	50	hsa-miR-205-5p	SYNPO2	synaptopodin 2

A target gene list of hsa-miR205-5p by the miRDB database. The target score of ZEB1 is 91, indicating a very high confidence prediction in being a target of hsa-miR205-5p
